# N-Cadherin Is Critical for the Survival of Germ Cells, the Formation of Steroidogenic Cells, and the Architecture of Developing Mouse Gonads

**DOI:** 10.3390/cells8121610

**Published:** 2019-12-11

**Authors:** Rafal P. Piprek, Michal Kolasa, Dagmara Podkowa, Malgorzata Kloc, Jacek Z. Kubiak

**Affiliations:** 1 Department of Comparative Anatomy, Institute of Zoology and Biomedical Research, Jagiellonian University, Gronostajowa 9, 30-387 Krakow, Poland; dagmara.podkowa@uj.edu.pl; 2 Institute of Systematics and Evolution of Animals, Polish Academy of Sciences, 31-018 Krakow, Poland; kolasa@isez.pan.krakow.pl; 3 The Houston Methodist Research Institute, Houston, TX 77030, USA; MKloc@houstonmethodist.org; 4 Department of Surgery, The Houston Methodist Hospital, Houston, TX 77030, USA; 5 MD Anderson Cancer Center, University of Texas, Houston, TX 77030, USA; 6 Cell Cycle Group, Faculty of Medicine, Institute of Genetics and Development of Rennes, UMR 6290 CNRS, Université de Rennes, F-35000 Rennes, France; jacek.kubiak@univ-rennes1.fr; 7 Laboratory of Regenerative Medicine and Cell Biology, Military Institute of Hygiene and Epidemiology (WIHE), 01-163 Warsaw, Poland

**Keywords:** N-cadherin, cell adhesion, testis, ovary, gonad development, testis cords, Sertoli cells, Leydig cells, interstitium, germ cells

## Abstract

Normal gonad development assures the fertility of the individual. The properly functioning gonads must contain a sufficient number of the viable germ cells, possess a correct architecture and tissue structure, and assure the proper hormonal regulation. This is achieved by the interplay between the germ cells and different types of somatic cells. N-cadherin coded by the *Cdh2* gene plays a critical role in this interplay. To gain an insight into the role of N-cadherin in the development of mouse gonads, we used the Cre-loxP system to knock out N-cadherin separately in two cell lines: the SF1^+^ somatic cells and the OCT4^+^ germ cells. We observed that N-cadherin plays a key role in the survival of both female and male germ cells. However, the N-cadherin is not necessary for the differentiation of the Sertoli cells or the initiation of the formation of testis cords or ovigerous cords. In the later stages of gonad development, N-cadherin is important for the maintenance of testis cord structure and is required for the formation of steroidogenic cells. In the ovaries, N-cadherin is necessary for the formation of the ovarian follicles. These results indicate that N-cadherin plays a major role in gonad differentiation, structuralization, and function.

## 1. Introduction

During sexual differentiation of the gonads, the testis cords or the ovigerous cords emerge from the gonad primordia, which are the amalgams of different cell types. In developing testis cords, the germ cells become enclosed by differentiating Sertoli cells (male supporting cells), and the interstitium forms between the cords. In developing ovaries, analogically, the germ cells become enclosed by the follicular cells to form the ovigerous cords, which later become divided into ovarian follicles. The stroma (an equivalent of the interstitium) is present between the ovigerous cords and the follicles [1]. The follicular cells (female supporting cells enclosing the germ cells in the ovaries) are homologs of the Sertoli cells [2]. Sertoli and follicular cells, i.e., supporting cells, differentiate from the steroidogenic factor 1 (SF1)-positive coelomic epithelium which covers developing gonads [2]. The coelomic epithelium also gives rise to some of the interstitial cells [1,3]. However, it is still unclear how, depending on the genetic sex, the cells of different types segregate and assemble to form the divergent structures.

It is known that cell adhesion plays a critical role in cell segregation and assembly during tissue and organ development. A cell of a given type has a specific set of cell adhesion molecules (CAMs) on its surface. This specific cell adhesion signature allows cells to recognize and adhere to each other. We demonstrated previously that CAMs are important for gonad differentiation, and that homologous cell types of different sexes differ in their CAM signature [4]. Additionally, we showed that the knockout of E-cadherin in the somatic or germ cells of developing gonads leads to germ cell loss [5]. The N-cadherin (Neural-cadherin, cadherin 2) is another well-studied adhesion molecule which is present at the surface of the somatic cells of undifferentiated gonads. In the differentiating ovaries, N-cadherin is present in all somatic cells, both stromal and follicular. In differentiating testes, N-cadherin disappears from the interstitial cells but remains at the surface of Sertoli cells [6,7]. N-cadherin participates in the formation of the adherens junctions (AJs), and promotes adhesion of same-type cells owing to homotypic interactions [8]. It is known that N-cadherin is important for the establishment and maintenance of the multicellular structure of organs [8]. Numerous studies have been devoted to the role of N-cadherin in adult testes, especially in spermatogenesis, sperm release, and formation of the blood–testis barrier [9,10,11], but no data are available regarding its specific role during testis development. The conditional knockout of N-cadherin was analyzed in Sertoli cells of adult mouse testes and the meiotic germ cells [12]. Because the expression pattern of N-cadherin varies between cell types and phases of gonad development [13], we wanted to verify our hypothesis that N-cadherin is involved in the tissue architecture of the gonads in early gonadogenesis, sexual differentiation of the gonads, and the formation of testis cords and ovarian follicles. It was particularly intriguing to us that N-cadherin expression augments in the somatic cells of developing gonads, which suggests the potential importance of N-cadherin for gonad development. The conventional knockout of N-cadherin, which shuts down N-cadherin expression in all cells, is lethal to embryos in the mouse; embryos die just before the onset of gonadogenesis, at E10 (embryonic day 10), due to the malformations of many organs [14]. To circumvent this problem, we performed the knockout of the N-cadherin coding gene using the Cre-loxP system where *Cre* recombinase is expressed under the promoters specific for the somatic or the germ cells. *Sf1* gene (steroidogenic factor 1, *Nr5a1*) expression starts in the coelomic epithelium (somatic) cells at the onset of the gonadal primordium formation. Thus, we used *Cre* recombinase driven by the expression of *Sf1* promoter to induce early deletion of N-cadherin in the somatic cells, which form the testis cords and interstitium [15]. To analyze the effects of N-cadherin knockout in the germ cells of developing gonads, we used *Cre* recombinase under the expression of *Oct4* (octamer-binding transcription factor 4, *Pou5f1*), which is a marker for germ cells in both sexes [16].

The knockout gonad phenotypes were studied using light and electron microscopy and immunohistochemistry. The expression of male sex determination genes (*Sox9*, *Amh*) and female sex determination genes (*Rspo1*, *Wnt4*) was analyzed by RT-qPCR to determine if a disruption of cell adhesion influences sex determination, which can lead to sex reversal (manifested as an imbalanced sex ratio, and an increased expression of male sex determination genes in XX gonads, and conversely, female sex determination genes in XY gonads). We also studied the expression of the markers for fetal Leydig cells (*Cyp11a1*, *Cyp17a1*), Sertoli cells (*Sox9*, *Amh*, *Dhh*), germ cells (*Mvh*, *Tra98*), proliferation (PCNA) and apoptosis (caspase 3) by RT-qPCR and immunohistochemistry.

## 2. Materials and Methods

### 2.1. Animals

The study was approved by the I Local Commission for Ethics in Experiments on Animals. The animals were bred and housed in the Animal Facility at the Jagiellonian University (Krakow, Poland). Three transgenic mouse lines used in this study were purchased from The Jackson Laboratory (ME, USA).

The mouse strain Tg(Nr5a1-cre)7Lowl/J has Cre recombinase expression in SF1-positive somatic cells of the gonads [15]. The strain B6(SJL)-Pou5f1^tm1.1(cre/Esr1*)Yseg^/J expresses Cre recombinase in the OCT4-positive germ cells after the induction (injection) with tamoxifen [16]. Pregnant mice were injected with tamoxifen (2 mg/40 g body mass dissolved in sunflower oil) three times: once per day on E10.5, E11.5, and E12.5. The injection of oil alone served as a control [5]. The strain B6.129S6(SJL)-*Cdh2^tm1Glr^*/J, in which exon 1 is flanked with *loxP* sequences was used to study the effect of the deletion of the *Cdh2* gene that encodes N-cadherin [17]. The Cre^+^,loxP^+/+^ and Cre^-^,loxP^fl/fl^ mice were used as a control. Individuals with the knockout in the somatic cells were referred to as sKO, and in the germ cells as gKO.

Timed matings were performed by placing one male with two females overnight. The following morning, females were checked for the presence of the vaginal plug, and the initial time of pregnancy was referred to as E0.5. Females were euthanized by spinal dislocation at embryonic days: E10.5, 11.5, 12.5, 13.5, 14.5, 16.5, 18.5, and the newborns were euthanized at 1 and 2 dpp (days post partum), as previously described [5].

### 2.2. Genotyping

The sex of all studied animals was established by genotyping using primers for *Sly* (Y chromosome) and *Xlr* (X chromosome). Primers used for genotyping of Cre^+^ and LoxP^+^ animals are listed in Supplementary Table S2. A standard PCR protocol was used for genotyping as previously described [5,18].

### 2.3. RNA Isolation from the Gonads and Real-Time Quantitative PCR

RNA isolation was performed as previously described [5]. Gonads from mouse fetuses and newborns from the same experimental group were pooled according to the sex and developmental stage. Total RNA was isolated using TRI Reagent (Merck, Darmstadt, Germany) and further purified with RNeasy Mini kit per manufacturer’s instructions (Qiagen, Valencia, CA, USA). Total RNA in RNase-free water was frozen at –80 °C and then used for multigene qPCR analysis. A total 50 ng RNA of each sample was reverse transcribed into cDNA using random primers and SuperScript III Reverse Transcriptase (ThermoFisher Scientific, Warsaw, Poland) following the manufacturer’s instructions. A list of primers is presented in Supplementary Table S2. The primers were designed using Primer Express™ Software v3.0.1 and provided by the Genomed Company (Warsaw, Poland). The RT-qPCR procedure in 5 μL reactions using SYBR Green Master Mix (ThermoFisher Scientific, Warsaw, Poland) and 200 nM concentration of each primer, and melting-curve analysis were performed in the 7500 Fast Real-Time PCR System (ThermoFisher Scientific, Warsaw, Poland) and High Resolution Melt (HRM) Software v2.0. We used PCR cycle conditions: 50 °C for 2 min (one cycle), 95 °C for 10 min (one cycle), 95 °C for 15 s and 60 °C for 1 min (35 cycles). Data were collected as raw C_T_ values and analyzed using the 2^−ΔΔ CT^ method. *Actb* and *Rn18S* were used as reference genes [19]. Gene expression was normalized on an arbitrary scale with reference gene as 1.0. Statistical analysis was performed using the nonparametric ANOVA Kruskal–Wallis test followed by the Tukey’s test. Statistica 7.0 software was used for the analyses.

### 2.4. Gonadal Cell Isolation and Sorting

To check the effectiveness of the knockout, we analyzed the expression of *Cre* and *Cdh2* genes separately in isolated SSEA1-positive germ cells and protocadherin 18 (PCDH18)-positive somatic cells, as previously described [5]. We previously also showed that *Pcdh18* is highly expressed in the supporting and interstitial/stromal cells, but not in the germ cells of developing mouse gonads of both sexes [13]. The Stage-specific embryonic antigen-1 (SSEA1) is a marker of germ cells previously used for isolation of undifferentiated germ cells from embryonic and adult gonads [20,21]. The gonads were dissected at E10.5, E11.5, E13.5, E16.5, 2 dpp, and were incubated in 250 μL 0.25% Trypsin–EDTA (Merck, Darmstadt, Germany) at 37 °C for 5 to 10 min as previously described [13]. After tissue dissociation, the enzyme solution was replaced with 400 μL PBS. Cells were centrifuged for 10 min at 10,000 rpm, and the cell pellet was resuspended in 3% BSA/PBS containing antibodies (10 μg/mL DyLight650-conjugated anti-SSEA1, Invitrogen, MA1-022-D650, and 10 μg/mL FITC-conjugated anti-PCDH18, Biorbyt, orb3038) and incubated for 30 min at RT. Subsequently, cells were centrifuged for 10 min at 10,000 rpm and resuspended in PBS. For cell sorting the MoFlo XDP cytofluorimeter with a sorter (Beckman Coulter, Indianapolis, IN, USA) was used. Cells were sorted depending on DyLight 650 and FITC signal. The cells were collected into lysis buffer (DNA/RNA Shield, Zymo Research R1100). Pooled gonads from three fetuses were used for each time point and experiments were repeated three times. Total RNA was isolated using TRI Reagent (Sigma, 93289), purified with RNeasy Mini kit according to the manufacturer’s instructions (Qiagen, Valencia, CA, USA), and RT-qPCR was performed as described above. To verify the expression of N-cadherin, we used primers designed for exon 1 of *Cdh2* mRNA, which is deleted in knockout individuals [17]. A list of primers is provided in Supplementary Table S1. The purity of isolated cells was confirmed by analysis of the expression of germ cell markers (*Dazl* and *Mvh*) and somatic cell markers (*Sox9*, *Foxl2*) (Supplementary Figure S1). The number of animals used for real-time qPCR analysis is presented in Supplementary Table S2.

### 2.5. Histology and Immunohistochemistry

Dissected gonads were fixed in Bouin’s solution, dehydrated, and embedded in paraffin (Paraplast, Sigma, P3683). Histological staining was performed according to Debreuill’s trichromatic (aniline blue and picric acid, Sigma, B8563) method as previously described [22,23]. For immunochemistry, heat-induced epitope retrieval was conducted in a sodium citrate buffer (10 mM sodium citrate, 0.05% Tween-20, pH 6) at 95 °C for 20 min as previously described [5]. Subsequently, the sections were blocked with 3% H_2_O_2_ and 10% goat serum (Sigma, G9023), incubated with primary antibodies (all rabbit polyclonal: anti-AMH, 1:100, Santa Cruz Biotechnology, sc-166752; anti-cleaved caspase 3, 1:100, Assay BioTech, L0104; anti-collagen I, 1:300, Abcam, ab34710; anti-Cre, 1:3000, Abcam, ab190177; anti-CYP17A1, 1:200, Abcam, ab125022; anti-N-cadherin, 1:1000, Abcam, ab76057; anti-PCNA, 1:500, Abcam, ab18197; anti-TRA98, 1:500, Abcam, ab82527) at 4 °C overnight, and stained using UltraVision Quanto Detection System (ThermoFisher, Fremont, CA, USA, TL-125-QHD). Mayer’s hematoxylin (Sigma, MHS32-1L) was used as a counterstain. Sections were viewed under the Nikon Eclipse E600 microscope. The number of animals used for histological and immunohistochemical analysis is presented in Supplementary Table S3.

### 2.6. Electron Microscopy

Dissected gonads were fixed in Karnovsky’s fixative, rinsed in cacodylate buffer and postfixed in 1% osmium tetroxide solution [24,25]. After dehydration in ethanol and acetone, samples were embedded in Epon 812 (Sigma, 45345), cut into semi-thin sections (0.5 μm), and stained with methylene blue (Sigma, 77515) and Azure (Sigma, A6270). Selected fragments were cut for ultra-thin sections (80 nm) and stained with uranyl acetate and lead citrate. The sections were analyzed with a JEOL JEM2100HT transmission electron microscope (Jeol Ltd., Tokyo, Japan).

### 2.7. Measurements 

A diameter of the testes, testis cords, and the ovaries was measured in the five widest cross-sections stained with Trichrome. The number of TRA98-positive germ cells, CYP17A1-positive fetal Leydig cells, PCNA-positive proliferating cells, and caspase 3-positive apoptotic cells was calculated within the 10,000 μm^2^ area in five cross sections from each gonad using ImageJ software. Average values and standard deviation of gonad size and cells numbers were calculated. The number of cells in the knockout gonads was compared to the control using the χ2 test. Statistical data were analyzed with Statistica 6 PL Software (Krakow, Poland).

## 3. Results

### 3.1. The Effectiveness of Genetic Knockout

To assess the effectiveness of the genetic knockout we first analyzed the level of *Cre* recombinase expression separately in the germ and somatic cells isolated from XY and XX mouse gonads. We detected the expression of *Cre* recombinase under the control of *Sf1* promoter from E11.5 onward in the PCDH18^+^ cells (somatic cells) isolated from both XY and XX gonads (Supplementary Figure S2A,B). Immunolocalization of Cre protein showed positive signal in the somatic cells of the testis cords and interstitial cells in XY gonads and the somatic cells in XX gonads, however, the control was negative for Cre protein (Supplementary Figure S2C–F). Additionally, there was a drop of *Cdh2* expression in PCDH18^+^ cells from E11.5 in both XY and XX gonads (Supplementary Figure S2G,H). Immunolocalization of N-cadherin showed lower signal in the knockout and control gonads comparing to control (Supplementary Figure S3A–D). 

High expression of the *Cre* gene under the control of *Oct4* promoter was detected in SSEA1^+^ cells (germ cells) isolated from embryonic XY and XX gonads from E11.5 onward (Supplementary Figure S4A,B). Immunolocalization of Cre protein showed positive signal in the germ cells in both XY and XX gonads, however, the control was negative for Cre protein (Supplementary Figure S4C–F). The expression of *Cdh2* was vestigial at E13.5, and absent at E16.5 (Supplementary Figure S4G,H), which indicated the effectiveness of the knockout of *Cdh2* in the germ cells. Due to the high expression of *Cdh2* in somatic cells enclosing germ cells, a signal of N-cadherin immunolocalization was only slightly lower in knockout gonads comparing to control (Supplementary Figure S3E–H).

### 3.2. Knockout of N-Cadherin (Cdh2) in SF1^+^ Somatic Cells of Developing Testis

**Figure 1:**
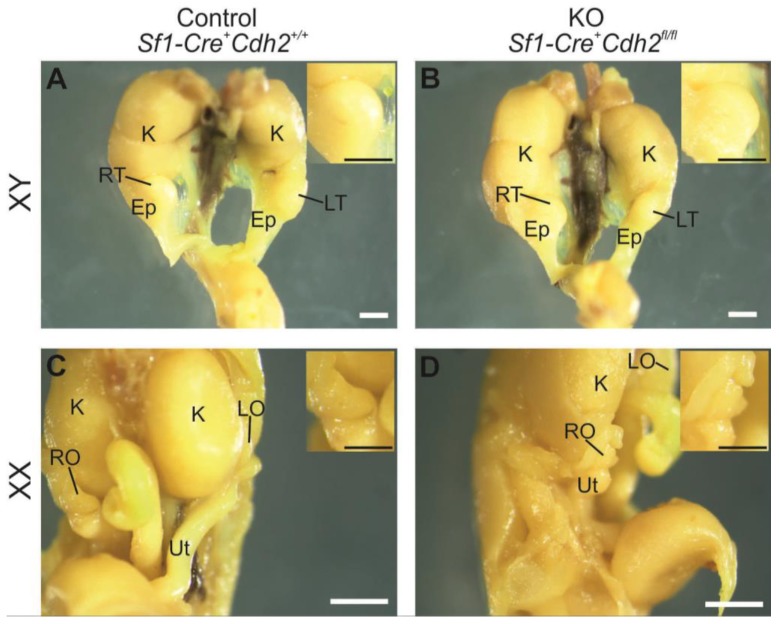
Morphology of the urogenital system of the control male (**A**), knockout male (**B**), control female (**C**), and knockout female (**D**) at E18.5. Ep—epididymis, K—kidney, LO—left ovary, LT—left testis, RO—right ovary, RT—right testis, Ut—uterus. Insets show magnified gonads. Stage: E18.5, scale bar is equal to 500 μm.

**Figure 2:**
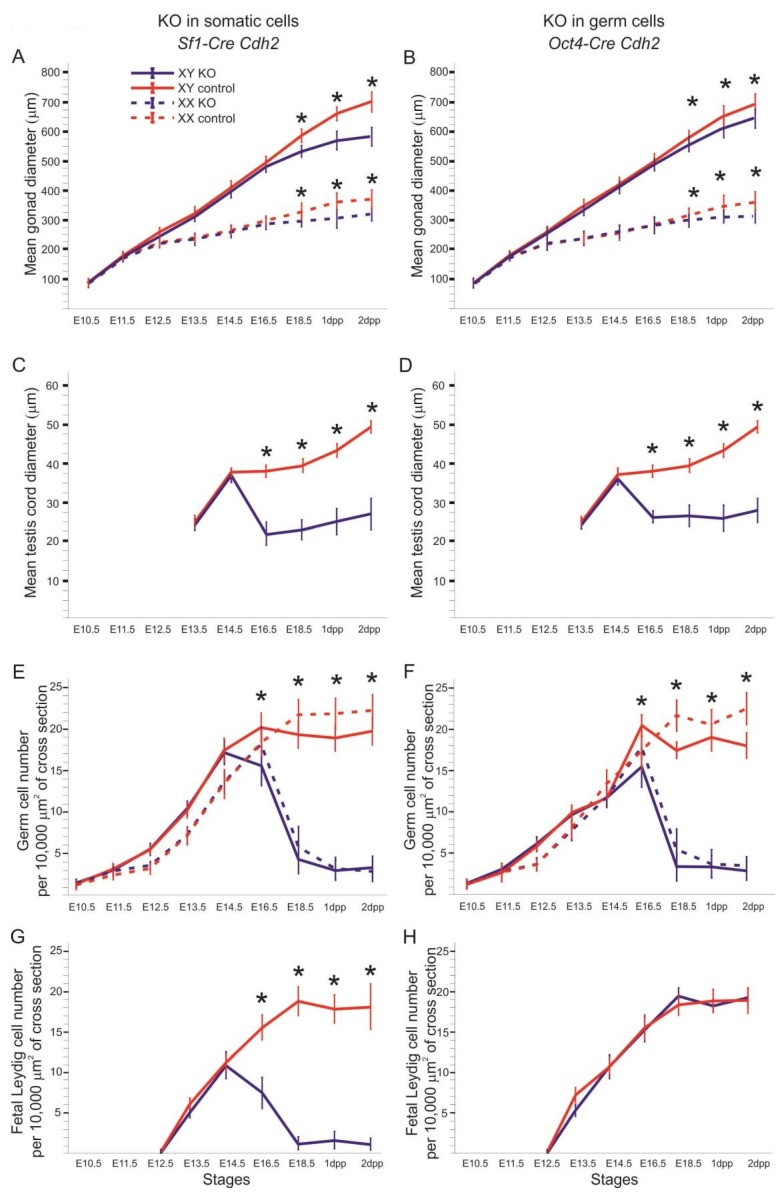
Diagrams of gonad size and cell number. (**A**) The mean of gonad diameter at consecutive stages of development in the *Cdh2* knockout in the SF1^+^ cells. (**B**) The mean of gonad diameter at consecutive stages of development in the *Cdh2* knockout in the OCT4^+^ cells. (**C**) The mean of testis cord diameter at consecutive stages of development in the *Cdh2* knockout in the SF1^+^ cells. (**D**) The mean of testis cord diameter at consecutive stages of development in the *Cdh2* knockout in the OCT4^+^ cells. (**E**) The number of germ cells in the *Cdh2* knockout in the SF1^+^ cells. (**F**) The number of germ cells in the *Cdh2* knockout in the OCT4^+^ cells. (**G**) The number of fetal Leydig cells in the *Cdh2* knockout in the SF1^+^ cells of XY gonads. (**H**) The number of fetal Leydig cells in the *Cdh2* knockout in the OCT4^+^ cells of XY gonads. * *P <* 0.05 (by χ^2^), deviation bars indicate standard deviations.

**Figure 3:**
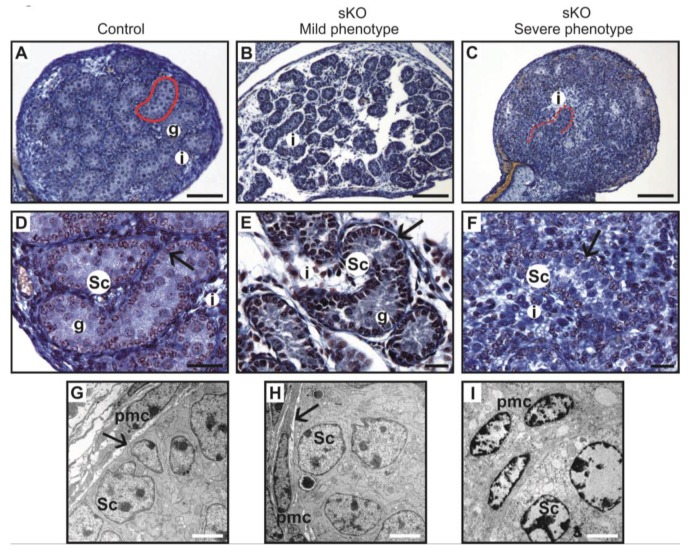
Histology of testis from the control and the N-cadherin knockout in the somatic cells. Trichrome staining: (**A**,**D**) Testes of the control individual (*Sf1-Cre^+^ Cdh2^+/+^*); germ cells (g) are enclosed by the Sertoli cell epithelium (Sc); the whole testis cord (encircled) is encapsulated by the basement membrane (arrows); the interstitium (i) is located between the cords. (**B**,**E**) The mild phenotype of the testis in the knockout individual (*Sf1-Cre^+^ Cdh2^fl/fl^*); the germ cells (g) are less numerous; continuous basement membranes (arrows) completely underlay epithelium of Sertoli cells. (**C**,**F**) The severe phenotype of the knockout (*Sf1-Cre^+^ Cdh2^fl/fl^*); testis cords are hardly distinguishable, and their borders are discontinuous (dotted line); within the aggregate of cells occasionally a fragment of Sertoli cells epithelium is discernible (Sc). Transmission electron microscopy: (**G**) The testis cord in the control testis; Sertoli cells (Sc) are enclosed by the basement membrane (arrow), peritubular myoid cells (pmc) are located around the cords. (**H**) The testis cord in the knockout testis with mild phenotype shows a similar structure to the control. (**I**) The disrupted testis structure in the knockout with severe phenotype shows the aggregate of cells (presumably Sertoli and peritubular myoid cells) without any basement membrane. Stage: E18.5, scale bar in panels (**A–C**) is equal to 100 μm, in panels (**D–F**) to 25 μm, and in panels (**G**–**I**) to 3 μm.

Upon analyzing the male phenotypes, we noticed the smaller dimensions of the testes in the newborn knockout males in comparison to the control (Figure 1A,B and Figure 2A). Histological analysis demonstrated that the control testes had thick testis cords tightly packed within the gonad (Figure 3A,D, left panel). Testis cords were composed of the germ cell aggregates enclosed by a monolayer of Sertoli cells (Figure 3D). The cords were surrounded by the basement membrane and the peritubular myoid cells (PMCs) (Figure 3D,G). The narrow spaces between the cords denoted the interstitium (Figure 3D).

**Figure 4:**
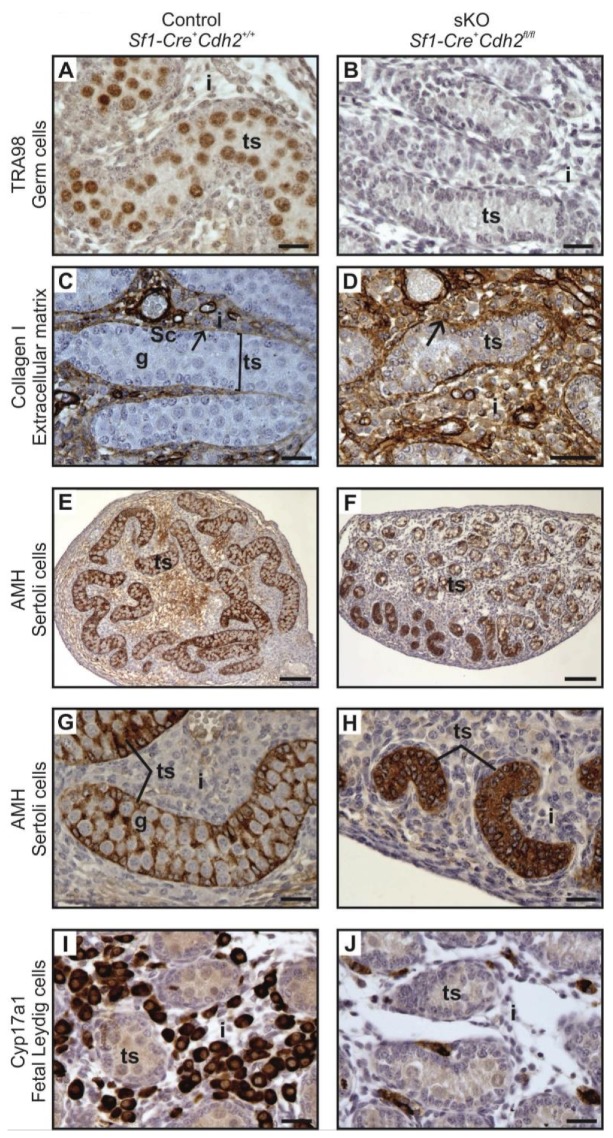
Immunostaining of control and N-cadherin knockout in the somatic cells. (**A**,**B**) TRA98 immunostaining shows numerous germ cells in the control testes, and no germ cells in the knockout testis. (**C**,**D**) Collagen I immunostaining in control and knockout testis. Regular oval testis cords (ts) are present in the control and irregular, smaller cords are present in the knockout testes. The cords are enclosed by the basement membrane (arrow), Sertoli cells (Sc), germ cells (g). Collagen I-positive interstitium (i) is located between the cords. (**E**–**H**) Anti-Müllerian hormone (AMH) immunostaining shows structure of the Sertoli epithelium; (**E**,**G**) In the control testes, the Sertoli cells (Sc) have many protrusions, which surround the germ cells (g). (**F**) The testis cords in the knockout testis have disrupted structure. (**H**) The testis cords in the knockout testis lack the germ cells—the whole cords are composed exclusively of Sertoli cells (stained). (**I**,**J**) Immunostaining for steroidogenesis enzyme—CYP17A1, shows numerous steroidogenic cells (fetal Leydig cells) in the interstitium of the control testis, while only a few CYP17A1 positive cells in the interstitium of the knockout testis (**J**). Stage: E18.5, scale bar in panels (**A**–**D**), **(G**–**J**) is equal to 25 μm and in panels (**E,F**) to100 μm.

In the N-cadherin (*Cdh2*) knockout in the SF1^+^ gonadal somatic cells (*Sf1-Cre^+^ Cdh2^fl/fl^*), the structure of the developing testis remained unaltered in comparison to the control until E16.5. After this stage, the testes of the knockout fetuses and newborns exhibited various degrees of structural changes. The changes in the morphology of testis cords in the knockout testes were mild or severe. The number of germ cells was significantly lower in the knockout testes starting from E16.5 than in control testes (Figure 2E, Figure 3B,C,E,F and Figure 4A,B). The germ cell loss was also reflected in the lower level of expression of germ cell marker *Mvh* in the knockout testes and the smaller dimension of testis cords (Figure 2C, Supplementary Figure S5A).

Electron microscopy study showed that the control and mild phenotype had well-defined testis cords (Figure 3G,H) with the germ cells enclosed by a continuous layer of Sertoli cells, well-developed basement membrane, and the peritubular myoid cells (PMCs) surrounding the cords. Testes with the severe phenotype had significant defects in the testis cord architecture. Due to the disruption of the basement membrane, the testis cords were locally “dissolved”, the integrity of Sertoli cell epithelium was disrupted, the Sertoli cells were scattered, and only occasionally the germ cells were present between the impaired cords (Figure 3C,F,I). Electron microscopy analysis revealed that in the severe phenotype, the somatic and germ cells formed disorganized masses of unlayered cells, and the basement membrane was discontinuous (locally absent) (Figure 3I).

To visualize the internal structure of the gonads and the outlines of testis cords, basement membrane, and interstitium, we performed immunostaining with anti-collagen I antibody. In the control, the shape of testis cords was regular (Figure 4C). In the knockout animals, the outline of numerous testis cords was irregular and folded (Figure 4D). Because some individuals showed disrupted integrity of the Sertoli cell epithelium (Figure 4E–H) we investigated the immunolocalization of the anti-Müllerian hormone (AMH), which is a Sertoli cell marker. In the control animals, the Sertoli cells formed a monolayer, had basally located nuclei and many thin protrusions, which enclosed the germ cells inside the testis cords (Figure 4E,G). In the knockout testes, because of the lower number of germ cells or their complete absence, the testis cords were smaller. The nuclei of Sertoli cells were tightly packed and the cell protrusions were missing. In addition, the centers of the testis cords were filled with the Sertoli cells (Figure 4F,H). Thus, in the knockout testes, the inner structure of testis cords was always altered. We observed two types of defective cords. In the first type, the cells did not adhere to one another resulting in the presence of free space between the Sertoli cells (Figure 4F). The second type contained the Sertoli cell-only cords in which only Sertoli cells with strongly stained cytoplasm were present (Figure 4F,H). These two types of defective cords were usually co-present in the same gonad (Figure 4F). Table 1 summarizes the effects of N-cadherin knockout in the somatic cells on the testes structure.

### 3.3. N-Cadherin Knockout in SF1^+^ Somatic Cells Affects Steroidogenic Fetal Leydig Cells (FLCs)

The N-cadherin knockout not only affected the structure of testis cords but also the intercord interstitium, which contains steroidogenic fetal Leydig cells (FLCs). The immunolocalization of cytochrome CYP17A1—a marker of FLCs—showed a significant decrease of FLC number in the developing testes of knockout mice (Figure 2G and Figure 4I,J). This decrease in the number of FLCs started at stage E16.5. In addition, the qPCR showed a decrease in the level of mRNA of another FLC marker—the *Cyp11a1* (Supplementary Figure S5A). These results indicate that the N-cadherin knockout affected the FLCs (Table 1).

### 3.4. The Effect of N-Cadherin Knockout in Somatic Cells on Sex Determination

The WNT4 and RSPO1 signaling molecules, acting via stabilization of β-catenin, play a pivotal role in the female sex determination pathway [26]. Because the cadherins, which are linked to the cytoskeleton via catenins, including β-catenin, interact with WNT/β-catenin pathway, we checked if the N-cadherin knockout influenced mouse sex determination. We used RT-qPCR to study the changes in the expression of sex determination genes (testis-specific: *Amh*, *Sox9*, *Dhh*, and ovary-specific: *Rspo1*, *Wnt4*, *Foxl2*), which are expressed in the gonadal somatic cells derived from the coelomic epithelium. We found that the level of expression of *Amh*, *Sox9,* and *Dhh* genes in the knockout testes was similar to the control (Supplementary Figure S5A). The testis-specific, but not ovarian-specific, genes were expressed in XY gonads. This indicated that the expression of sex-determining genes agreed with the genetic sex of the individual and that the deletion of N-cadherin did not cause sex reversal, and thus did not influence sex determination.

### 3.5. Knockout of N-Cadherin (Cdh2) in SF1^+^ Somatic Cells of the Developing Ovary

**Figure 5:**
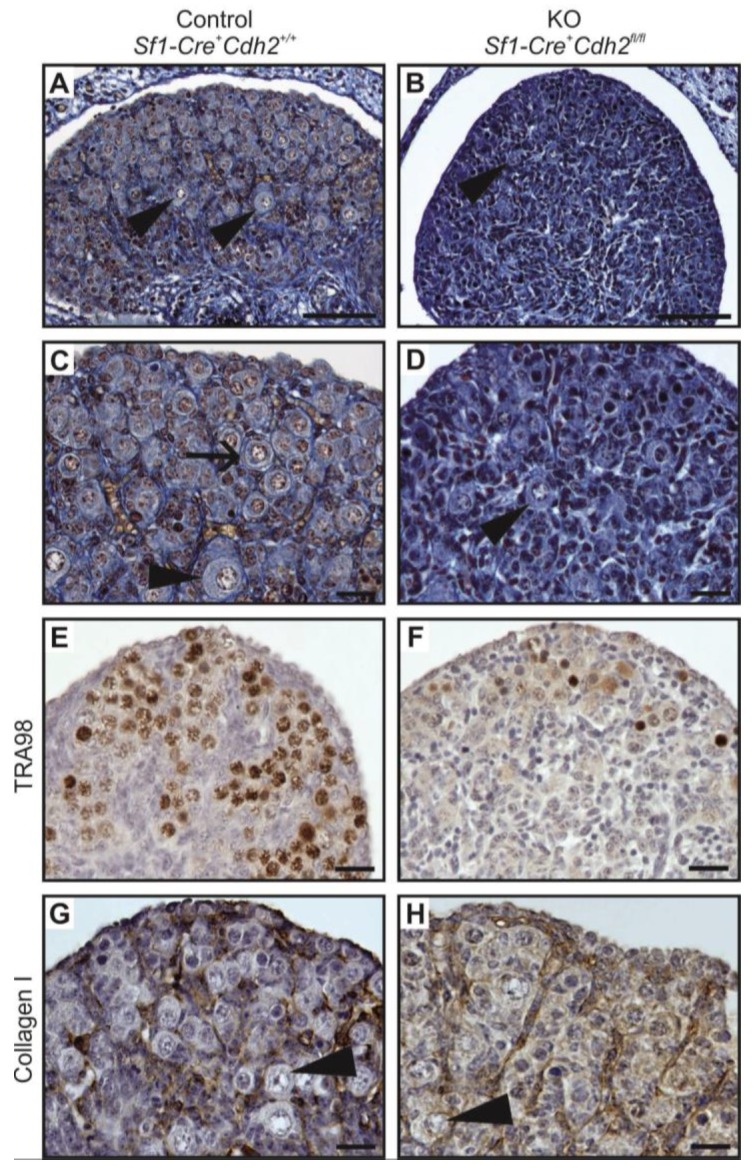
Structure of the ovary of the control and the N-cadherin knockout in the somatic cells. (**A**,**C**) Histology of the control ovary (*Sf1-Cre^+^ Cdh2^+/+^*) shows numerous germ cells, including oocytes (arrowheads) surrounded by the follicular cells (arrow) in the ovarian cortex. (**E**) TRA98 staining shows numerous germ cells. (**G**) Collagen I immunostaining marks the basement membranes dispersed between groups of cells. (**B**,**D**) Histology of knockout ovary (*Sf1-Cre^+^ Cdh2^fl/fl^*) shows only a few oocytes (arrowhead). (**F**) The TRA98 immunostaining reveals the presence of only a few germ cells (**H**). Stage: E18.5, in panels (**A**,**B**) the scale bar is equal to 100 μm and in (**C**–**F**) to 25 μm.

The SF1^+^ knockout ovaries had lower mean diameter than the control, and the structure of the knockout ovaries was different than in the control (Figure 1C,D, Figure 2A and Figure 5A–D). The most striking feature of the knockout phenotype was a dramatic decrease in the number of germ cells (Figure 2E and Figure 5F). Accordingly, the level of expression of *Mvh*—a germ cell marker—was much lower in N-cadherin knockouts (Supplementary Figure 5B).

The histological analysis of the female gonads revealed that the ovigerous cords of control ovaries contained numerous germ cells enclosed by somatic cells (Figure 5A,C,E,G, left panel). The ovigerous cords were surrounded by the collagen I-positive basement membrane (Figure 5G). In the N-cadherin knockout ovaries, due to the lack or very low frequency of germ cells, the ovigerous cords were composed of the rows of somatic cells, and their basement membrane was positive for collagen I (Figure 5H). This indicates that the lack of N-cadherin did not disrupt the formation of ovigerous cords. However, no ovarian follicles were observed in the knockout ovaries even at 2 dpp. Table 2 summarizes the effects of N-cadherin knockout in the somatic cells on the ovaries. We also did not detect any significant differences in the expression of the ovarian-specific genes (*Rspo1*, *Wnt4*, *Foxl2*) between the control and knockout ovaries (Supplementary Figure S5B).

### 3.6. Knockout of N-Cadherin (Cdh2) in OCT4^+^ Germ Cells in Developing Testes

**Figure 6:**
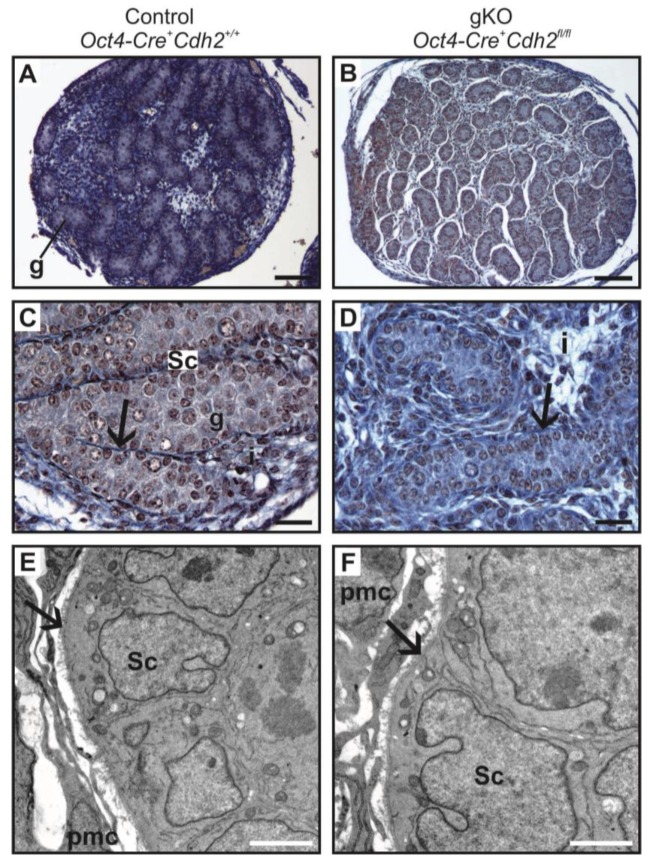
Histology of testis of the control and the N-cadherin knockout in the germ cells. (**A**,**C**) Testes from the control individual (*Oct4-Cre^+^ Cdh2^+/+^*); numerous germ cells (g) are enclosed by epithelium composed of the Sertoli cells (Sc); the testis cords are encapsulated by the basement membrane (arrows); the interstitium (i) is located between the cords. (**B**,**D**) Testis in the knockout individual (*Oct4-Cre^+^ Cdh2^fl/fl^*); only a few germ cells (g) are present; the continuous basement membranes (arrows) underlay Sertoli epithelium. Transmission electron microscopy: (**E**) The testis cord in the control testis (*Oct4-Cre^+^ Cdh2^+/+^*); Sertoli cells (Sc) are enclosed by the basement membrane (arrow), peritubular myoid cells (pmc) are located around the cords. (**F**) The testis cord in the knockout testis (*Oct4-Cre^+^ Cdh2^fl/fl^*) has the structure similar to the control. Stage: E18.5, in panels (**A,B**) the scale bar is equal to 100 μm; in panels (**C,D**) to 25 μm and in panels (**E,F**) to 2 μm.

**Figure 7:**
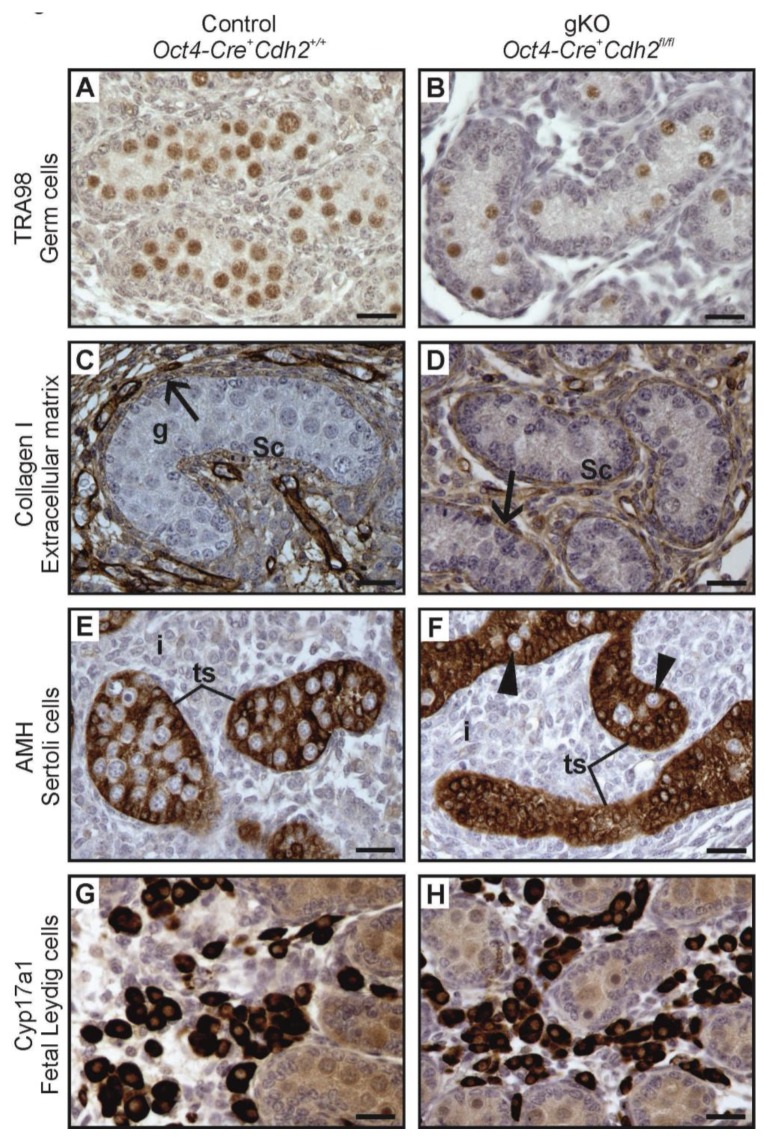
Immunostainings of the control and the N-cadherin knockout in the germ cells. (**A**,**B**) The TRA98 immunostaining shows numerous germ cells in the control testis (*Oct4-Cre^+^ Cdh2^+/+^*), and a lower number of germ cells in the knockout testis (*Oct4-Cre^+^ Cdh2^fl/fl^*). (**C**,**D**) The collagen I immunostaining in the control and knockout testis shows testis cords with Sertoli epithelium (Sc), germ cells (g) and continuous basement membranes (arrows), with no significant differences between the control and knockout testis cords. (**E**) Immunostaining for anti-Müllerian hormone (AMH) in the control testis shows normal morphology of testis cords (ts) and Sertoli cells with many protrusions enclosing the germ cells. (**F**) Immunostaining for AMH in the knockout testis shows the aggregates of Sertoli cells filling the entire cords (tc) and only a few germ cells (arrowheads). (**G**,**H**) Immunostaining for CYP17A1 shows no differences in the number of fetal Leydig cells between the control and the knockout testis. Stage: E18.5, scale bar is equal to 25 μm.

The knockout of N-cadherin in the germ cells (*Oct4-Cre^+^ Cdh2^fl/fl^*) resulted in the lower mean diameter of testes, lower mean diameter of testis cords, decreased number of the germ cells and reduced level of expression of *Mvh* (germ cell marker) in comparison to the control (Figure 2B,D,F, Figure 6A–D and Figure 7A,B; Supplementary Figure S5C). Similar to the knockout of N-cadherin in the somatic cells, the germ cell loss started being visible around E16.5 (Figure 2F).

Even in the case of a complete lack of the germ cells, the testes cords had normal Sertoli cell epithelium surrounded by the basement membrane (Figure 6D,F, and Figure 7B,D,F). The phenotype of testis cords in the germ cell knockout was similar to the mild phenotype knockout in the somatic cells (Figure 6D,F). The collagen I immunostaining showed undisrupted integrity of the testis cords (Figure 7C,D). AMH immunostaining showed that the testis cords contained only Sertoli cells, and the germ cells were present only sporadically (Figure 7E,F). Table 3 summarizes the effects of N-cadherin knockout in the germ cells on the testes.

Gene expression analysis of Sertoli markers (*Amh*, *Sox9,* and *Dhh*) did not show any significant difference between the knockout and the control (Supplementary Figure S5C). The CYP17A1 immunostaining showed no significant difference in the number of FLCs between the knockout and control testis (Figure 2H and Figure 7G,H). In contrast to the N-cadherin knockout in the somatic cells, the knockout in the germ cells did not affect the level of *Cyp11a1* mRNA (Supplementary Figure S5C).

### 3.7. Knockout of N-Cadherin (Cdh2) in OCT4^+^ Germ Cells in Developing Ovaries

**Figure 8:**
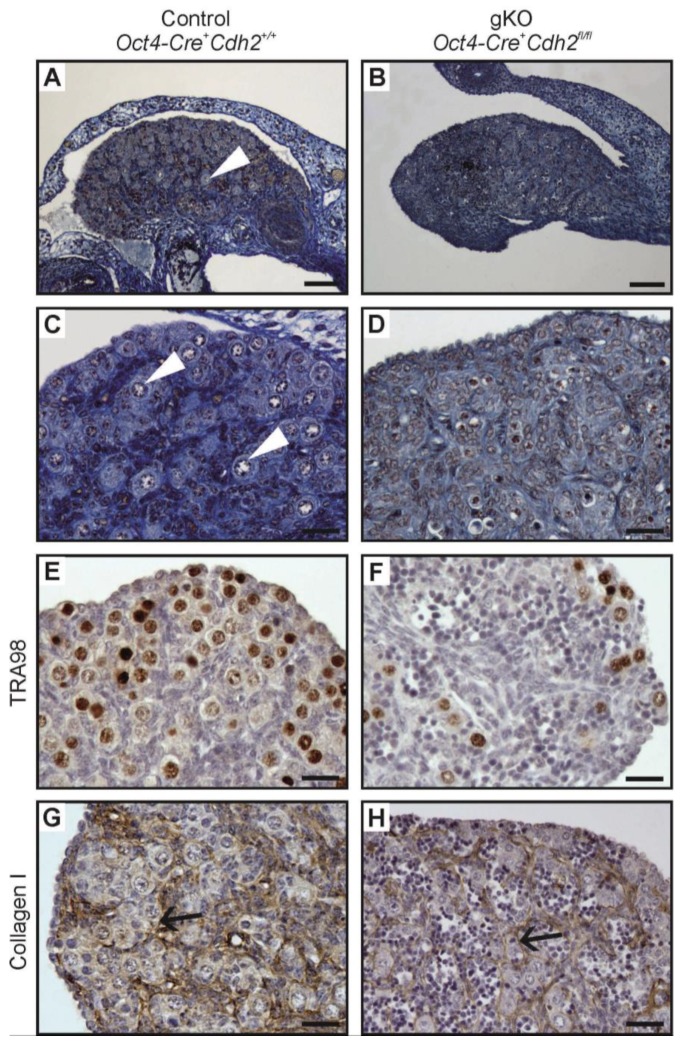
Structure of the ovary of the control and the N-cadherin knockout in the germ cells. Histology (**A**,**C**) of the control ovary (*Oct4-Cre^+^ Cdh2^+/+^*) with numerous oocytes (arrowhead). Histology (**B**,**D**) of the knockout ovary (*Oct4-Cre*^+^ *Cdh2^fl/fl^*) lacking the oocytes. (**E**,**F**) The TRA98 immunostaining shows numerous germ cells in the control ovaries and a lower number of germ cells in the knockout ovaries. (**G**,**H**) Immunostaining for collagen I in the control and knockout ovary shows basement membranes (arrows) outlining the ovigerous cords in both the control and the knockout. Stage: E18.5, scale bar in panels (**A**,**B**) is equal to 100 μm and in panels (**C**–**F)** to 25 μm.

The N-cadherin knockout in the germ cells in XX gonads resulted in a reduction of mean ovary diameter and the loss of germ cells (Figure 2B,D; Figure 8, right panel). The deposition of collagen I remained unchanged in the knockout ovaries (Figure 8H; Supplementary Figure S5D). The ovigerous cords were present, however, no ovarian follicles were present even at 2 dpp (Figure 8, right panel). The expression of the female sex-determining genes (*Rspo1*, *Wnt4*, *Foxl2*) remained unaltered in the knockout ovaries in comparison to the control (Supplementary Figure S5D). Table 4 summarizes the effects of N-cadherin knockout in the germ cells on the ovaries.

### 3.8. Proliferation and Apoptosis in N-Cadherin Knockouts 

**Figure 9:**
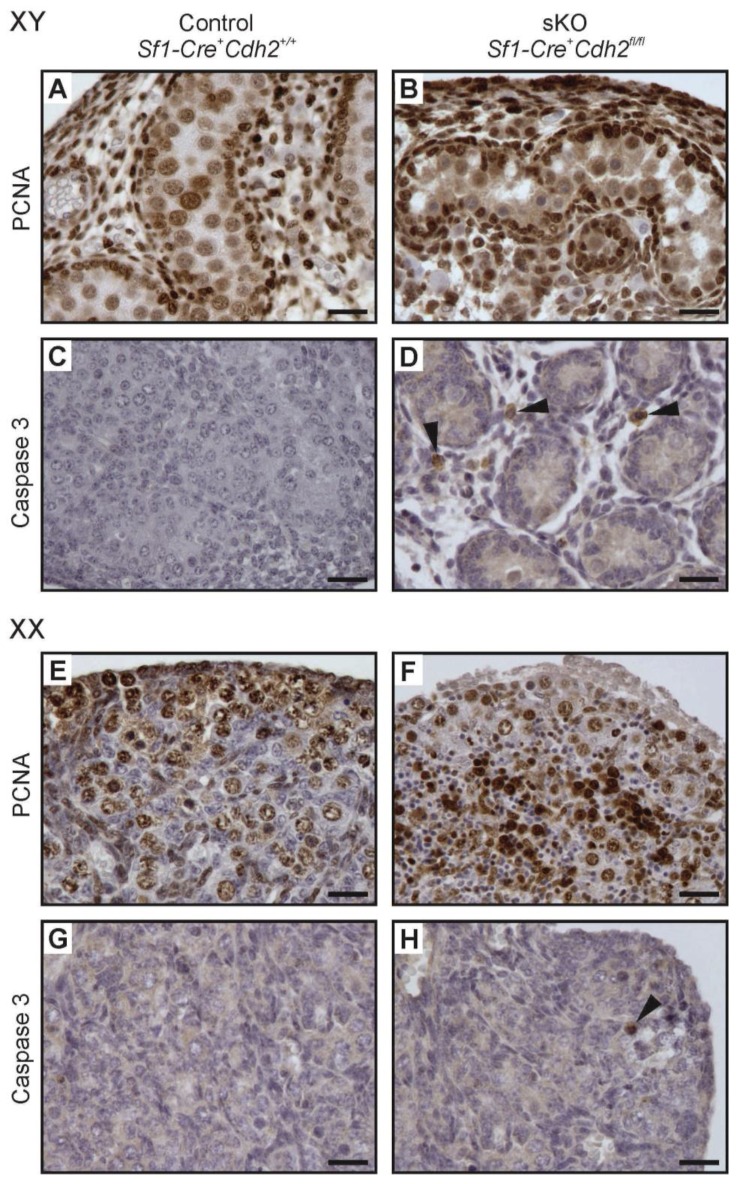
Proliferation (PCNA immunostaining) and apoptosis (caspase 3 immunostaining) in testes and ovaries of the N-cadherin knockout in the somatic cells (sKO). (**A**,**B**) PCNA immunostaining in control and knockout testis. (**C**,**D**) Caspase 3 immunostaining in control and knockout testis; there is a higher number of positive cells (arrowheads) in the knockout especially in the interstitium than in the control. (**E**,**F**) PCNA immunostaining in control and knockout ovary. (**G**,**H**) Caspase 3 immunostaining in control and knockout ovary. Stage: E18.5, scale bar is equal to 25 μm.

**Figure 10:**
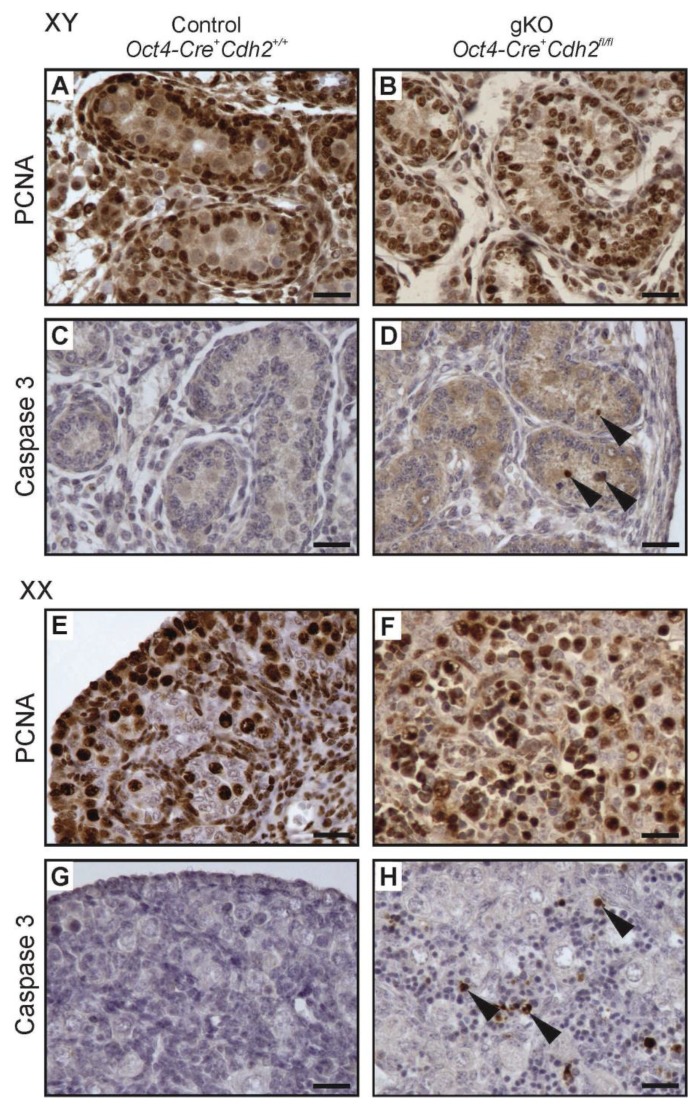
Proliferation (PCNA immunostaining) and apoptosis (caspase 3 immunostaining) in testes and ovaries of the N-cadherin knockout in the germ cells (gKO). (**A**,**B**) PCNA immunostaining in control and knockout testis. (**C**,**D**) Caspase 3 immunostaining in the control and knockout testis; many positive cells (arrowheads) are present in the knockout especially in the testis cords in comparison to the control. (**E**,**F**) The PCNA immunostaining in the control and knockout ovary. (**G**,**H**) Caspase 3 immunostaining in the control and knockout ovary. Stage: E18.5, scale bar is equal to 25 μm.

The N-cadherin deletion in the somatic or germ cells of developing mouse gonads caused the loss of germ cells. In addition, N-cadherin deletion in the somatic cells decreased the number of steroidogenic FLCs. Thus, we tried to determine if the defects in the gonad structure and the germ cell loss resulted from the changes in cell proliferation or apoptosis, two critical processes involved in cell and tissue homeostasis. We performed immunostaining with the proliferation marker (PCNA) and the apoptosis marker (caspase 3) antibodies (Figure 9 and Figure 10). The results of proliferation and apoptosis analysis are presented in Table 1, Table 2, Table 3 and Table 4. There was no significant difference in the number of PCNA-positive cells between control and knockout individuals of both sexes, which suggested that the defects in the structure of knockout gonads were not caused by the changes in cell proliferation (Figure 9A,B,E,F, and Figure 10A,B,E,F; Supplementary Figure S6). Caspase 3 immunostaining showed that the knockout testes and ovaries had significantly more apoptotic cells than control gonads (Figure 9C,D,G,H and Figure 10C,D,G,H; Supplementary Figure S6C,D). The testes with deleted N-cadherin in the somatic cells showed accumulation of apoptotic cells in the interstitium (Figure 9D), which may correspond to the FLC loss in the interstitium (Figure 4J), and inside the testis cords (Figure 10D), which possibly corresponds to the germ cell loss.

To show if there is a proliferation or apoptosis in the somatic or germ cells, we studied the expression of the proliferation marker *Ki-67* and apoptosis marker *Bax* in the somatic and germ cells. The expression of *Ki-67* did not differ between knockout and control cells (Supplementary Figures S7A,C,E,G, and S8A,C,E,G). However, the expression of *Bax* was higher in the germ cells in both knockouts (SF1-Cre^+^ and OCT4-Cre^+^), which correlated with the loss of germ cells, and increased caspase 3 immunostaining (Supplementary Figures S7B,D,F,H, and S8B,D,F,H). There was also an increase of the *Bax* expression in the somatic cells isolated from XY SF1-Cre^+^ knockouts, which correlated with the increased caspase 3 immunostaining in the interstitium (Supplementary Figure S7D).

## 4. Discussion

We showed previously that the conditional gene knockout of cell adhesion molecule E-cadherin (*Cdh1*) in mouse embryonic gonads in the SF1^+^ somatic cells or OCT4^+^ germ cells caused the loss of germ cells in both sexes and smaller gonad dimension without impaired tissue integrity [5]. Here we showed that the knockout of N-cadherin (*Cdh2*) showed a much stronger phenotype. It led not only to the loss of germ cells but also fetal Leydig cells, and affected the structure of the gonads, especially strongly manifested in the testes, which had significantly disorganized testis cords. This indicates that although N-cadherin seems to be equally important for the germ cell survival in testes and ovaries, it is more important for the development of testis structure, which is more complex than the structure of the ovary. Although the germ cell loss observed in N-cadherin knockouts was not surprising because it is known that the mutations of various genes involved in gonad development and gametogenesis lead to the loss of germ cells [27], our study suggests that the cell adhesion between either Sertoli or granulosa cells and their respective sex-specific germ cells is important for the survival of germ cells. We believe that the disruption of cell adhesion in N-cadherin knockouts caused changes in the intracellular signaling, which triggered germ cell apoptosis and resulted in germ cell loss. 

Several studies indicated that N-cadherin is involved in the anti-apoptotic signaling in various cell types. For example, early study showed that N-cadherin inhibits apoptosis of granulosa cells in rat ovaries [28]. A molecular mechanism explaining N-cadherin importance for cell survival is still poorly understood. It has been shown that overexpression of N-cadherin induces anti-apoptosis pathway (or prevents apoptosis) in cancer cells through upregulation of decoy receptor DCR2, which plays an inhibitory role in apoptosis, a downregulation of death receptor DR5, which mediates apoptosis, and through the interference with MAPK/ERK and NF-kB/p65 signaling [29]. N-cadherin was shown to upregulate Bcl2 (B-cell lymphoma 2), which encodes an anti-apoptotic factor in human prostate cancer cells [30]. N-cadherin also induces phosphatidylinositol-3 kinase (PI3K) dependent activation of Akt (protein kinase B), which enhances the Akt-cell survival pathway; thus N-cadherin inhibits apoptosis through the PI3K/Akt signaling pathway [30]. It was also proved that N-cadherin interacts with and stabilizes the FGF receptor 1 (FGFR1), which prevents apoptosis [31]. It has been shown that deletion of N-cadherin led to a decreased number of cardiac progenitor cells [32], and increased apoptosis in esophageal cancer cells [33]. The N-cadherin cell–cell dependent contacts promoted cell survival in human saphenous vain smooth muscles [34], and protected pancreatic beta cells from apoptosis [35]. The effect of N-cadherin on apoptotic pathway seems to be cell type-specific. For example, in the osteoblasts, N-cadherin negatively regulated cell survival via inhibition of PI3K/Akt signaling, and upregulation of apoptosis inducing Bax/Bcl2 mechanism [36]. Thus, N-cadherin is involved in development and carcinogenesis not only by creating cell–cell contacts, but also by acting as an important moderator of cell survival via several different, and cell-type specific anti-apoptotic signaling pathways. Further studies are required to resolve which signaling pathways are influenced by N-cadherin to regulate germ cell survival in developing gonads.

Several studies also indicated an anti-apoptotic function of E-cadherin. E-cadherin was shown to decrease apoptosis via regulation of Akt kinase activity in granulosa cells [37], blocking of E-cadherin induced apoptosis of murine keratinocytes [38], and loss of E-cadherin caused apoptosis of intestinal epithelial cells [39]. However, in human cells in vitro, a deletion of E-cadherin led to decreased apoptosis due to Notch-dependent upregulation of Bcl2 expression [40]. The epithelial cancer cell lines expressing higher E-cadherin levels displayed greater sensitivity to DR4/DR5-mediated apoptosis [41]. N-cadherin seems to have a different contribution to the regulation of cell survival than E-cadherin, which can explain the different effects of N- and E-cadherin knockouts on gonad development.

The clear difference in the phenotype of gonads developing under E-cadherin and N-cadherin knockout suggests a specific role of N-cadherin in the formation of the gonad structure, while either is equally needed for the normal germ cell survival. Alternatively, N-cadherin may be simply a major cadherin in the gonads (it is impossible to compare relative amounts of proteins by immunolocalization experiments), which would explain the stronger effect of its knockout. Interestingly, neither E-cadherin nor N-cadherin knockout triggers reciprocal overexpression. This suggests that there is no or only a very low transcriptional control correlation between these two genes. Previous studies documented the redundancy within cadherins, including the redundancy between N-cadherin and other cadherins [42,43,44,45,46,47,48]. To verify if the effects of N-cadherin knockout in our study could be compensated by the function of the other cadherins, we examined the expression of two other cadherins highly expressed in the developing gonads: E-cadherin (*Cdh1*) and P-cadherin (*Cdh3*) (Supplementary Figure S9). We showed that the expression of E-cadherin was much lower in the N-cadherin-knockout gonads than in the control, and the expression of P-cadherin was slightly lower. The E-cadherin is highly expressed in the germ cells, and a decrease of E-cadherin expression probably results from the germ cells loss in N-cadherin-knockout gonads. We did not find any increase in the expression of cadherin, which suggested that there is no redundancy among studied cadherins.

We observed that the knockout of N-cadherin in developing gonads, similar to the knockout of E-cadherin, did not cause the germ cell loss before stage E16.5 [5]. At present, it is unknown why the period preceding the E16.5 is critical for germ cell survival. In the ovaries, the majority of germ cells enter meiosis by E15.5 and some of the oocytes reach diplotene phase by E17.5 [49]. At this time, the germline cysts contain developing oocytes surrounded by pre-follicular cells (differentiating follicular cells) [50,51]. During the subsequent cyst breakdown, the pre-follicular cells surrounding the cysts penetrate inward and separate the oocytes into individual ovarian follicles. In this process, a correct contact and adhesion between adjacent germ cells and between germ and somatic cells play a very important role. When the cysts break down during normal development, the germ cells undergo apoptosis and only around 33% of the germ cells survive [52]. Our study showed that the presence of oocytes is required for the formation of the ovarian follicles; N-cadherin knockout ovaries with a lower germ cell number did not form the ovarian follicles.

In developing testes, around the birth, the prospermatogonia begin to relocate from the center of the cords toward their surface and gain contact with the basement membrane. At that time, spermatogonial stem cells differentiate. The ability of those cells to adhere to the basement membrane and build a niche is critical for the establishment of the spermatogonial stem cell pool. Impairment of cell adhesion during this relocation of prospermatogonia to the peripheral position in the cords results in their apoptosis (anoikis) [53]. We showed here that the impaired cell adhesion in N-cadherin knockouts caused germ cell death. This indicates that during the early stage of spermatogenesis, a proper adhesion between somatic and germ cells is critical for germ cell survival and maintenance. However, N-cadherin is not necessary for the postnatal entry of spermatogonia into meiosis [12]. Our experimental model, which allowed us to delete N-cadherin early during embryonic stages of gonadogenesis, showed that N-cadherin is important for germ cell survival around stage E16.5, i.e., just after prospermatogonia enter G0 in developing testes, or when the germline cysts break down in developing ovaries.

In the current study, we demonstrated that the knockout of N-cadherin in the germ cells caused their loss, which in turn resulted in the changes in the structure of the gonads (smaller gonads, smaller testis cords, and the lack of ovarian follicles). The N-cadherin knockout in SF1^+^ cells caused not only the germ cell loss but also profound changes in testes structure (disruption of testis cords). In the severe phenotype, the wall of testis cords and the basement membrane were disrupted. In contrast, the knockout of E-cadherin did not lead to a disruption of testis cords [5]. The lack of N-cadherin, but not E-cadherin, most likely resulted in a weakened adhesion between Sertoli cells. In the most severe phenotype, the Sertoli cells dispersed and the wall of the cords changed because of the lack of the basement membrane and the disrupted arrangement of the peritubular myoid cell layers. This indicates that the faulty adhesion between Sertoli cells can completely disrupt the testis structure. All these structural alterations did not prevent differentiation of Sertoli cells and male sex determination of the gonad. The level of expression of Sertoli cell markers (*Sox9*, *Amh*, *Dhh*) was unaffected. The knockout of E-cadherin also did not affect the expression of these markers [5].

Previously, the effects of N-cadherin knockout on spermatogenesis were analyzed in juvenile and adult mouse testes, where N-cadherin was deleted in differentiating Sertoli cells in already formed testis cords [12]. This study showed the defects during the progression of spermatogenesis, such as germ cell translocation from the peripheral to the adluminal position within seminiferous tubules beginning at 21 dpp, and the reduction in the number of meiotic cells and spermatozoa. More apoptotic cells were detected and presumably, this was the cause of lower fertility. Additionally, the giant multinucleated cells and vacuoles appeared in the seminiferous tubules starting at 80 weeks after birth. Also, the integrity of the blood–testis barrier (BTB) was affected indicating that N-cadherin is important for the proper function of Sertoli cells in the adult testes [12]. The authors did not report, however, any malformations of the testis cords. Thus, the presence of defective testis cords observed in our study most likely resulted from much earlier, i.e., during gonad development, deletion of N-cadherin.

We showed that the knockout of N-cadherin in the SF1^+^ gonadal somatic cells caused not only defects in testis cords but also in the interstitium. In contrast, the knockout of E-cadherin did not affect the interstitium [5]. DeFalco and coworkers (2011) showed that the interstitial cells derive from at least two distinct sources: SF1^+^ coelomic epithelium and SF1^-^ gonadal-mesonephros boundary [3]. In our experimental animals, the N-cadherin was deleted specifically in SF1^+^ cells. Because of this, only a subpopulation of interstitial cells was N-cadherin negative. We noticed two main features of interstitium defects: a dramatic reduction in the number of CYP17A1^+^ cells and a lower level of *Cyp11a1* mRNA in the knockout testes, which suggest impairment in FLC number and/or function. Hypothetically, these defects in the interstitial architecture and steroidogenesis could be an indirect effect of alterations of Sertoli cells, which secrete signaling molecules, such as desert hedgehog (DHH), critical for the differentiation of FLCs. However, we detected the normal level of *Dhh* expression in the knockout testes, which strongly suggests that the changes in the interstitium are not Sertoli cell-related, but rather they are a direct result of N-cadherin deletion in the interstitial cells.

It cannot be excluded that in our experiment the N-cadherin was also deleted in other tissues expressing *Sf1,* such as the hypothalamus and pituitary gland. Nevertheless, previous studies showed that deletion of gene encoding FSH (follicle-stimulating hormone, produced by the pituitary gland) did not influence gonad structure or the germ cell number, but rather caused the development of gonad tumors [54]. Similarly, a lack of LH (luteinizing hormone, another pituitary hormone) did not influence the development of mouse gonad but was crucial for stimulation of spermatogenesis during maturation [55]. This suggests that the phenotypes observed in our study were effects of N-cadherin deletion in the gonads and not in the hypothalamus or pituitary gland.

## 5. Conclusions

The presence of N-cadherin in germ and somatic cells of developing gonads is critical for the survival of the germ cells in both testes and ovaries. The presence of N-cadherin is not necessary for Sertoli cell differentiation and the formation of testis cords or ovigerous cords, and, thus, for sexual differentiation of the gonads. However, the presence of N-cadherin in the Sertoli cells is important for the maintenance of testis cord structure and N-cadherin in the ovaries is necessary for the development of follicular cells. The presence of N-cadherin in the interstitial cells is important for the establishment of a pool of steroidogenic cells (fetal Leydig cells).

## Figures and Tables

**Table 1:** Testes—the effects of N-cadherin sKO in the SF1^+^ somatic cells.

	Control	Mild Phenotype	Severe Phenotype
Testis size	Normal	Smaller	Smaller
Testis cord structure	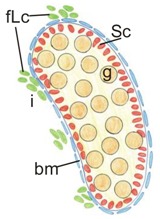	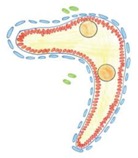	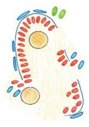
Testis cords	Normal, thick	Smaller, irregular shape	Disrupted
Germ cells (g)	Normal number of germ cells	Singular germ cells or no germ cells	Small number or no germ cells
Sertoli cells (Sc)	Continuous monolayer of Sertoli cells	Continuous monolayer of Sertoli cells	Disrupted monolayer of Sertoli cells
Basement membrane (bm)	Continuous	Continuous	Discontinuous
Fetal Leydig cells (fLc)	Normal number	Lower number	Lower number
Proliferation	Normal (high level)	Normal (high level)	Normal (high level)
Apoptosis	Normal (low level)	Increased	Increased

**Table 2:** Ovaries—the effects of N-cadherin sKO in the SF1^+^ somatic cells.

	Control	Knockout Phenotype
Ovary size	Normal	Smaller
Ovigerous cord structure	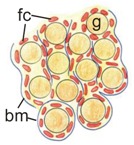	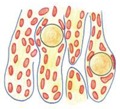
Ovigerous cords	Normal, irregular	Smaller, straight
Germ cells (g)	Normal number of germ cells	Lower number of germ cells
Follicular cells (fc)	Present	Present
Basement membrane (bm)	Continuous	Continuous
Proliferation	Normal (high level)	Normal (high level)
Apoptosis	Normal (low level)	Increased

**Table 3:** Testes—the effects of N-cadherin gKO in the OCT4^+^ germ cells.

	Control	Mutant Phenotype
Testis size	Normal	Smaller
Testis cords	Normal	Smaller
Germ cells	Normal number of germ cells	Lower number of germ cells
Sertoli cells	Continuous monolayer of Sertoli cells	Continuous monolayer of Sertoli cells
Basement membrane	Continuous	Continuous
Fetal Leydig cells	Normal number	Normal number
Proliferation	Normal (high level)	Normal (high level)
Apoptosis	Normal (low level)	Increased

**Table 4:** Ovaries—the effects of N-cadherin gKO in the OCT4^+^ germ cells.

	Control	Mutant Phenotype
Ovary size	Normal	Smaller
Ovigerous cords	Normal	Smaller
Germ cells	Normal number of germ cells	Lower number of germ cells
Follicular cells	Present	Present
Basement membrane	Continuous	Continuous
Proliferation	Normal (high level)	Normal (high level)
Apoptosis	Normal (low level)	Increased

## Supplementary Materials

The supplementary materials are available online at https://www.mdpi.com/2073-4409/8/12/1610/s1.

## Abbreviations

Actb—Beta-actin; Akt—Protein kinase B; Amh—Anti-Müllerian hormone; Bax/Bcl2—Bcl2-associated X protein; Bcl2—B-cell lymphoma 2; CAM—Cell adhesion molecule; Cdh1—Cadherin 1 (E-cadherin); Cdh2—Cadherin 2 (N-cadherin); Cre—Cre (causes recombination) recombinase; Cyp11a1—Cholesterol side-chain cleavage enzyme; Cyp17a1—Cytochrome P450 17A1; Dazl—Deleted in azoospermia-like; DCR2—Decoy receptor 2; Dhh—Desert hedgehog; dpp—days *post partum*; DR5—Death receptor 5; FGFR1—Fibroblast growth factor receptor 1; FLCs—Fetal Leydig cells; Foxl2—Forkhead box protein L; FSH—Follicle-stimulating hormone; Ki-67—Proliferation antigene Ki-67; LH—Luteinizing hormone; loxP—Locus of X(cross)-over in P1; MAPK/ERK—Mitogen-activated protein kinases/extracellular signal-regulated kinases; Mvh—Mouse vasa homologue; NF-kB/p65—Nuclear factor kappa-light-chain-enhancer of activated B cells; Oct4 (Pou5f1)—Octamer-binding transcription factor 4; Pcdh18—Protocadherin 18; PCNA—Proliferating cell nuclear antigen; PI3K—Phosphatidylinositol-3 kinase; Rspo1—R-spondin 1; SF1 (Nr5a1)—Steroidogenic factor 1; Sly—Sycp3-like Y-linked gene; Sox9—SRY-Box 9; SSEA1—Stage-specific embryonic antigen-1; Tra98—Germ cell-specific antigen; Wnt4—Wnt family member 4; Xlr—X linked, lymphocyte regulated
