# Supplementary material for: N-Cadherin Is Critical for the Survival of Germ Cells, the Formation of Steroidogenic Cells, and the Architecture of Developing Mouse Gonads
Source: Cells. 2019 Dec 11;8(12):1610. doi: 10.3390/cells8121610 (PMC6952792; doi:10.3390/cells8121610)
Supplement: Supplementary file 1 [file cells-08-01610-s001.pdf]

Suppl. Figure S1.

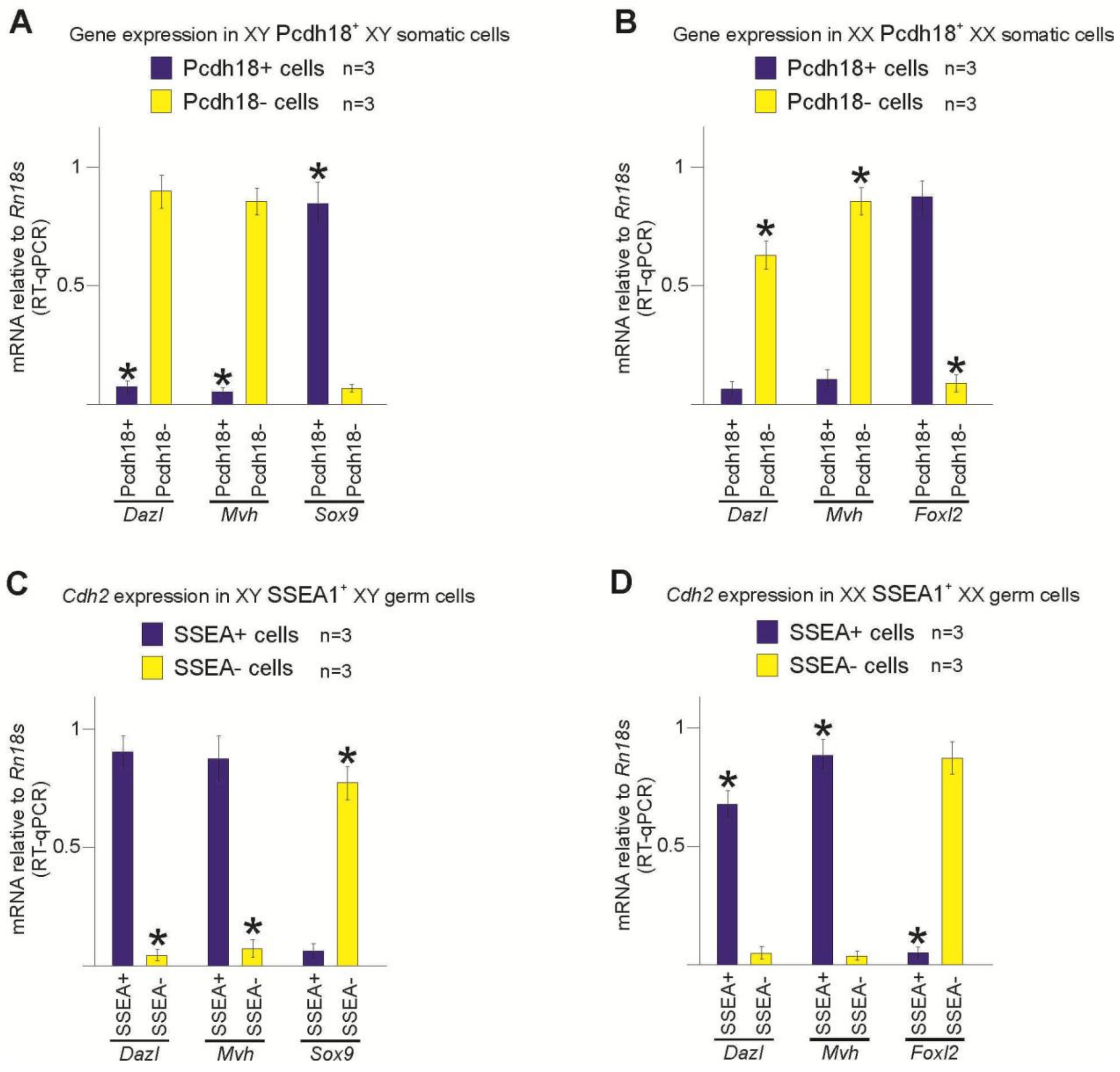

Supplementary Figure S1. Gene expression in FACS segregated cells. (**A**, **B**) Low expression of germ cell markers (*Oct4* and *Mvh*) and high expression of somatic cell marker (*Sox9*) in PCDH18$^+$ cells isolated from XY and XX gonads at E16.5 proves the purity of the isolated gonadal somatic cells. (**C**, **D**) Low expression of somatic cell marker (*Sox9*) and high expression of germ cell markers (*Oct4* and *Mvh*) in the SSEA1$^+$ cells isolated from XY and XX gonads at E16.5 proves the purity of the isolated germ cells. * $P<0.05$ (by χ^2), n – number of individuals tested, deviation bars indicate standard deviation.

Suppl. Figure S2.

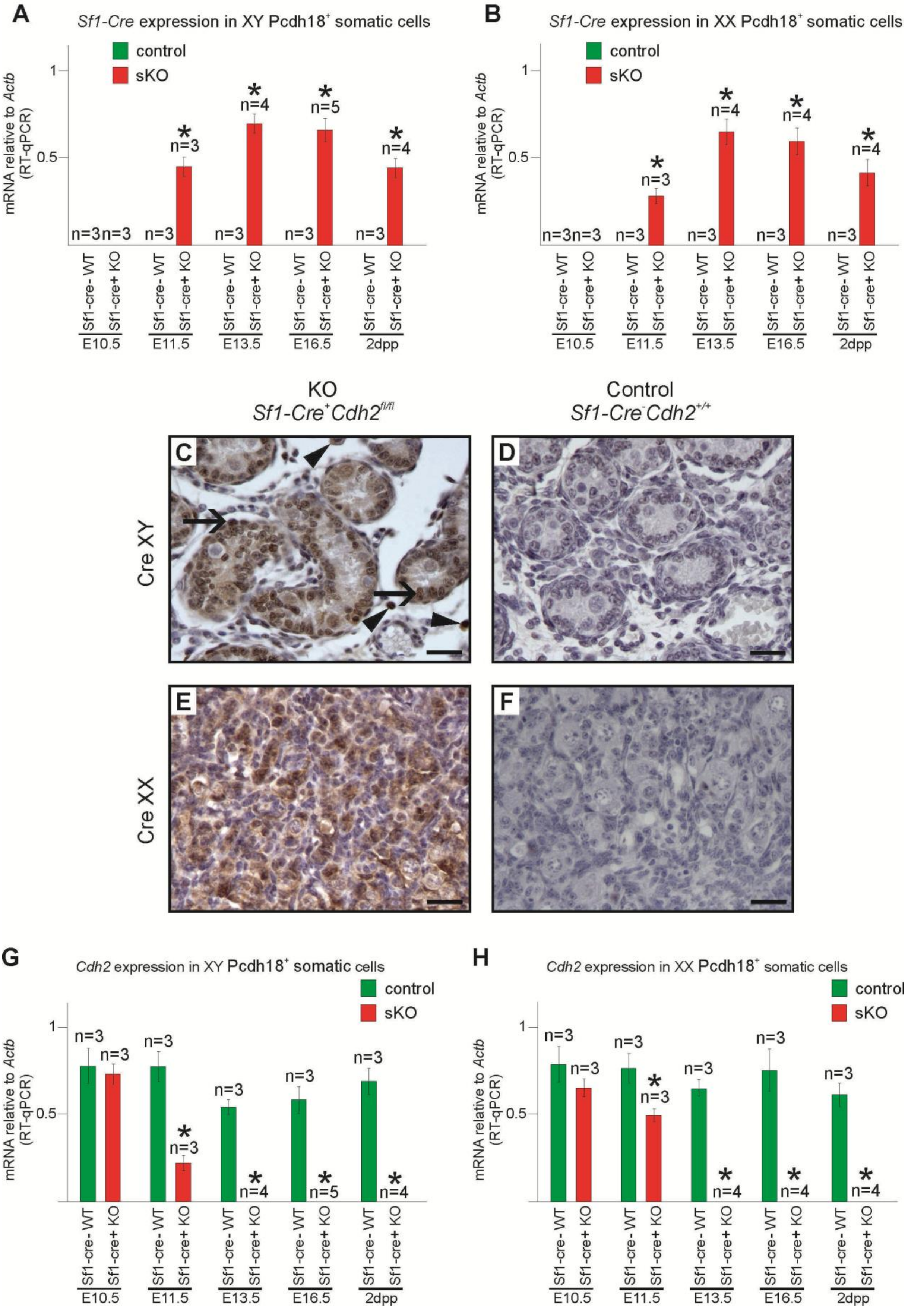

Supplementary Figure S2. Evaluation of *Cdh2* knockout effectiveness in the somatic cells. (**A**) The expression of *Cre* recombinase under *Sf1* promotes in PCDH18$^+$ cells isolated from the XY gonads. (**B**) The expression of *Cre* recombinase under *Sf1* promoter in the PCDH18$^+$ cells isolated from the XX gonads. In both XY and XX cells, the *Cre* expression was detectable from E11.5 onward, and no *Cre* expression was detected in the control. (**C**) Immunolocalization of Cre protein in the knockout testis at E18.5 – positive signal is present in somatic cells in the testis cords (arrows) and in the interstitium (arrowheads). (**D**) There is no signal of Cre protein in the control testis at E18.5. (**E**) Positive signal of Cre immunolocalization in the somatic cells of the knockout ovary at E18.5. (**F**) There is no signal of Cre protein in the control ovary at E18.5 (scale bar is equal to 25 μm). (**G**) The expression of *Cdh2* in the PCDH18$^+$ cells isolated from the XY gonads. The *Cdh2* expression was lower at E11.5, and absent from E13.5 onward. (**H**) The expression of *Cdh2* in the PCDH18$^+$ cells isolated from the XX gonads. The *Cdh2* expression was lower at E11.5, and absent from E13.5 onward. * $P<0.05$ (by χ^2), n – number of individuals tested, deviation bars indicate standard deviation.

Suppl. Figure S3.

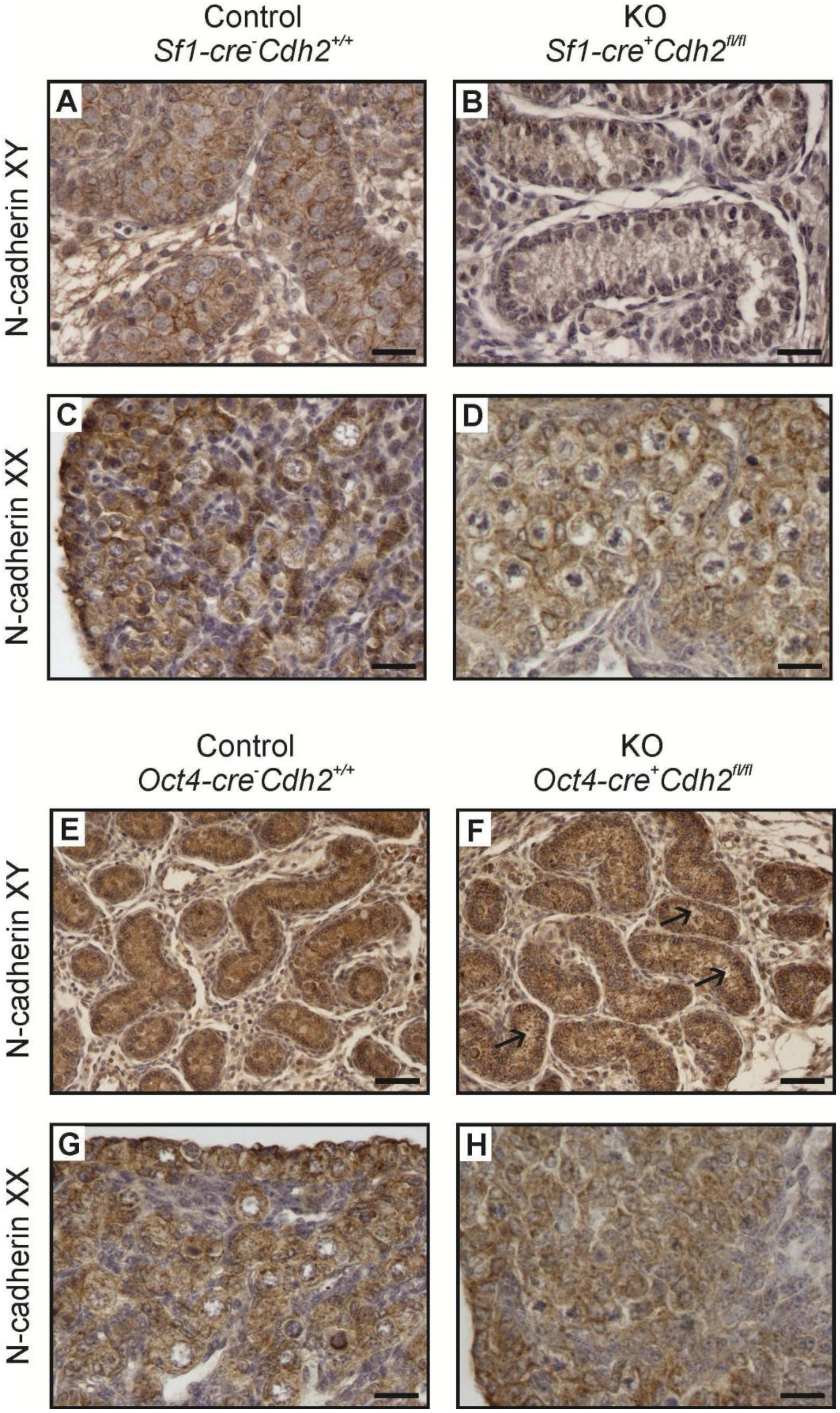

Supplementary Figure S3. Immunolocalization of N-cadherin. (**A**, **B**) The N-cadherin-positive signal is visibly lower in the knockout testis *Sf1-cre*$^{+}$*Cdh2*$^{fl/fl}$ when N-cadherin is deleted in somatic cells in comparison to the control. (**C**, **D**) The N-cadherin-positive signal is visibly lower in the knockout ovaries *Sf1-cre*$^{+}$*Cdh2*$^{fl/fl}$ in comparison to the control. (**E**, **F**) Pale germ cells (arrows) within testis cords indicate lower signal of N-cadherin immunostaining in testis with deleted N-cadherin in the germ cells (*Oct4-cre*$^{+}$*Cdh2*$^{fl/fl}$) in comparison to the control; a high signal of N-cadherin in the knockout testes results from the high expression of N-cadherin in the somatic cells. (**G**, **H**) The N-cadherin-positive signal is slightly lower in the knockout ovaries *Oct4-cre*$^{+}$*Cdh2*$^{fl/fl}$ when N-cadherin is deleted in the germ cells in comparison to the control. Stage: E18.5, scale bar is equal to 25 μm.

Suppl. Figure S4.

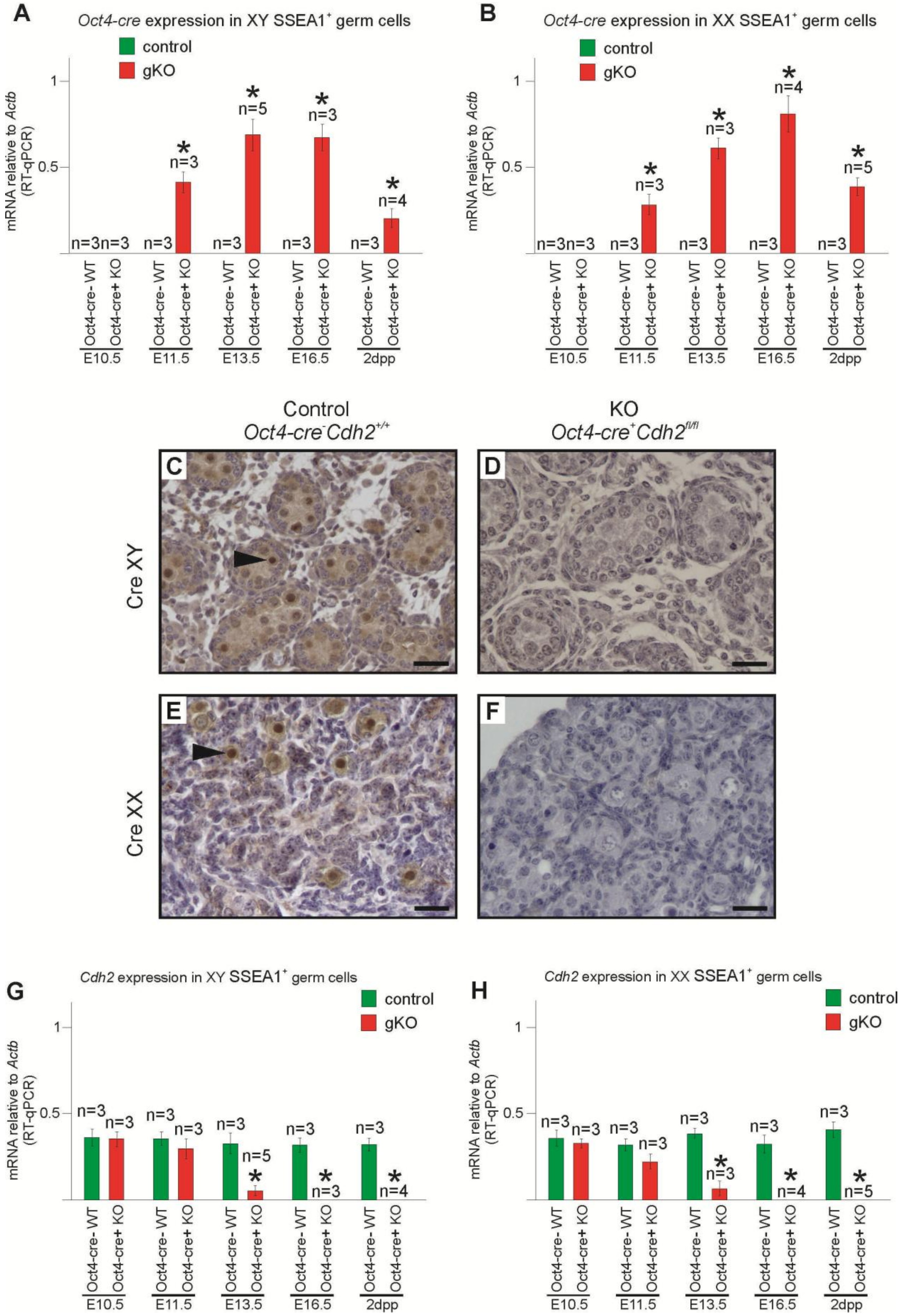

Supplementary Figure S4. Evaluation of the *Cdh2* knockout effectiveness in the germ cells. (**A**) The expression of *Cre* recombinase under *Oct4* promoter in the SSEA1$^+$ cells isolated from the XY gonads. (**B**) The expression of *Cre* recombinase under *Oct4* promoter in the SSEA1$^+$ cells isolated from the XX gonads. In both XY and XX cells, the *Cre* expression was detectable from E11.5 onward, and no *Cre* expression was detected in the control. (**C**) Immunolocalization of Cre protein in the knockout testis at E18.5 – positive signal is present in the germ cells (arrowhead). (**D**) There is no signal of Cre protein in the control testis at E18.5. (**E**) Positive signal of Cre immunolocalization in the germ cells of the knockout ovary at E18.5. (**F**) There is no signal of Cre protein in the control ovary at E18.5 (scale bar is equal to 25 μm). (**G**) The expression of *Cdh2* in the SSEA1$^+$ cells isolated from the XY gonads. The *Cdh2* expression was lower at E13.5, and absent from E16.5 onward. (**H**) The expression of *Cdh2* in the SSEA1$^+$ cells isolated from the XX gonads. The *Cdh2* expression was lower at E11.5, and absent from E13.5 onward. * $P<0.05$ (by χ^2), n – number of individuals tested, deviation bars indicate standard deviation.

Suppl. Figure S5.

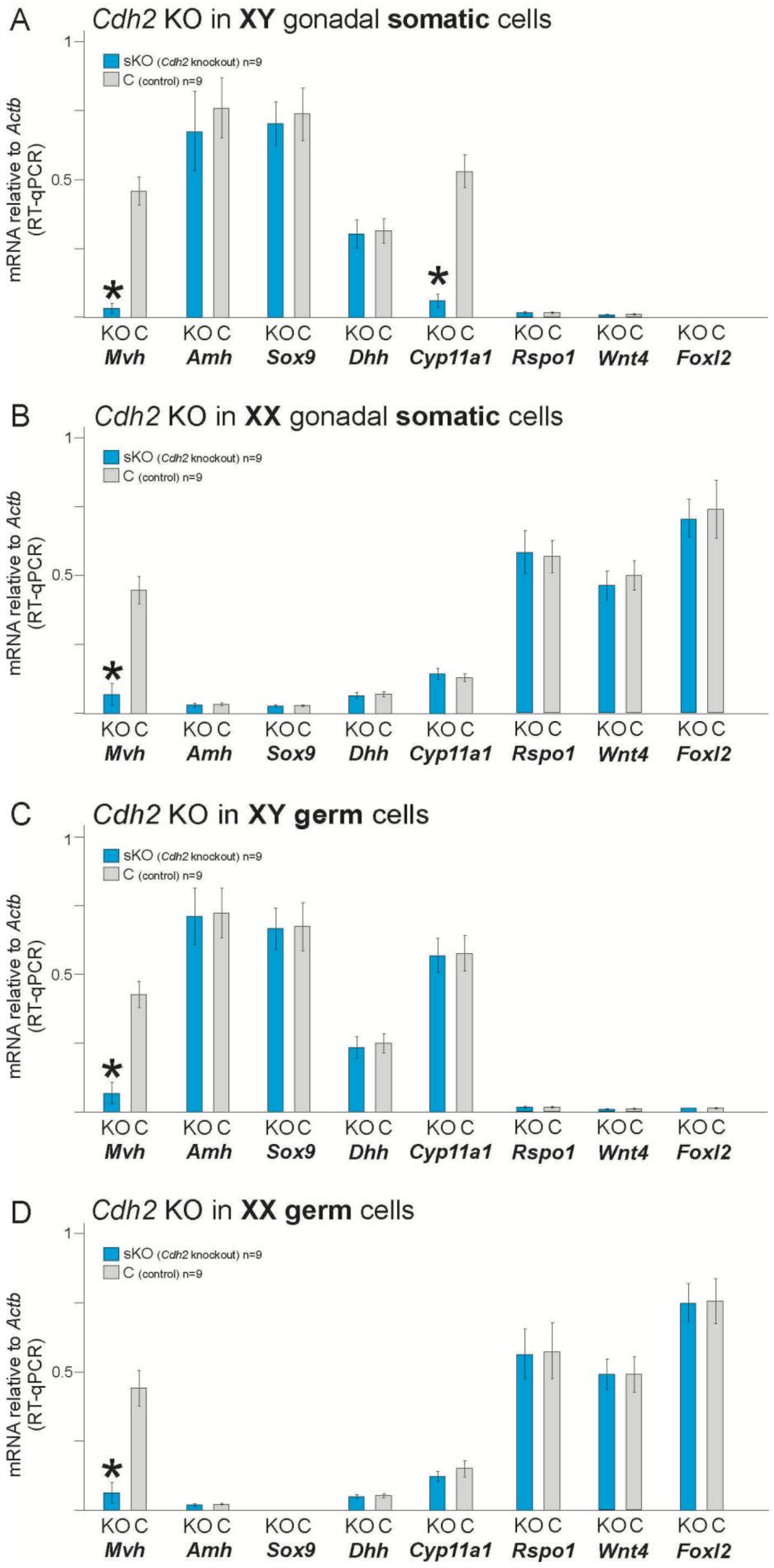

Supplementary Figure S5. Gene expression in the control gonads and the gonads with N-cadherin deleted in the XY gonadal somatic cells (A), in the control gonads and gonads with N-cadherin deleted in the XX gonadal somatic cells (B), in the control gonads and gonads with N-cadherin deleted in the XY germ cells (C), and in the control gonads and gonads with N-cadherin deleted in the XX germ cells (D). The expression of germ cell marker – *Mvh* is significantly decreased in all knockouts (**A**-**D**) in comparison to the control. The expression of steroidogenic enzyme *Cyp11a1* is significantly lower only in the XY gonads with N-cadherin deleted (**A**). The expression of Sertoli cell markers (*Amh*, *Sox9*, *Dhh*) and ovarian-differentiation markers (*Rspo1*, *Wnt4*, *Foxl2*) does not show significant differences between knockout and control gonads (**A**-**D**). Stage: E18.5, * $P<0.05$ (by χ^2), n – the number of individuals tested, deviation bars indicate standard deviation.

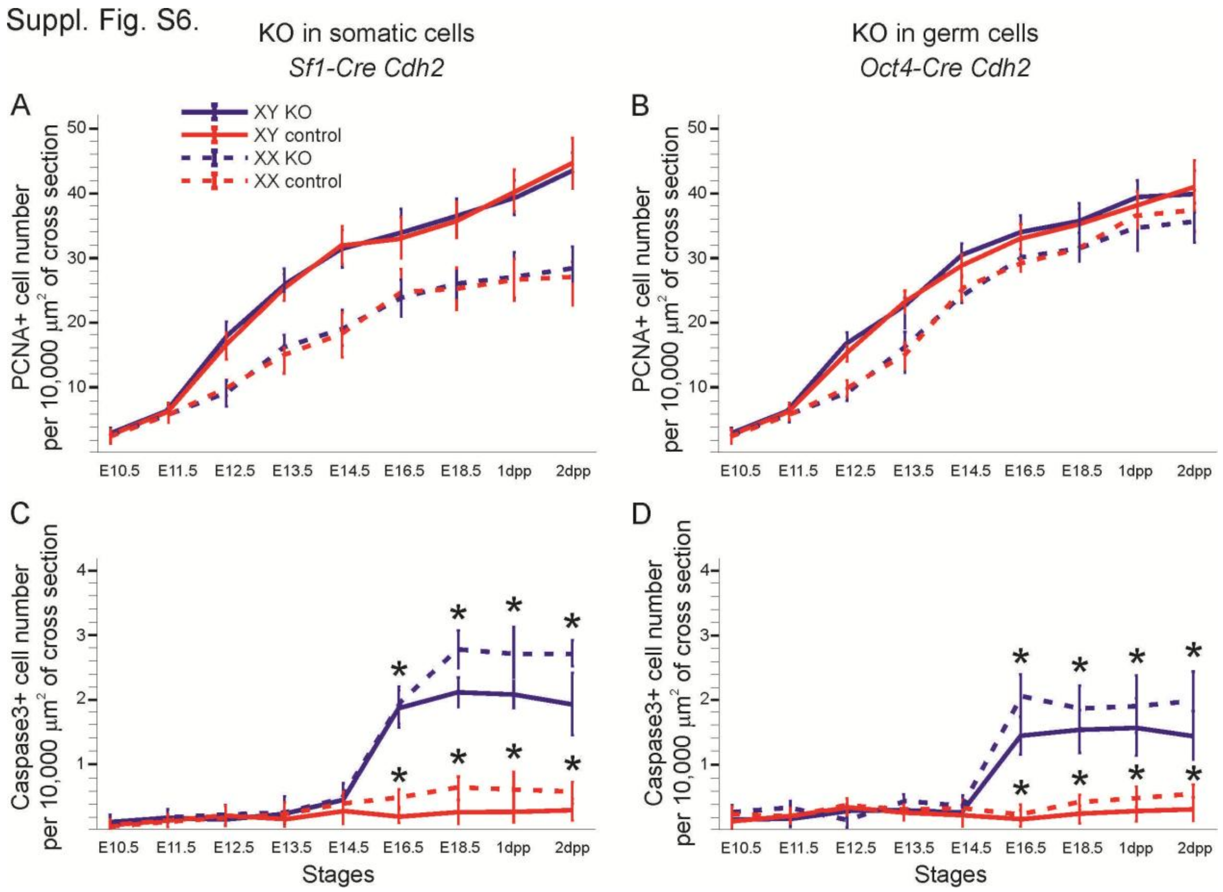

Supplementary Figure S6. The number of proliferating and apoptotic cells visible at cross-section. (**A**) There is no statistically significant difference between the number of PCNA+ cells in the control and N-cadherin knockout in the XY and XX gonadal somatic cells (sKO). (**B**) There is no significant difference between the number of PCNA+ cells in the control and N-cadherin knockout in the XY and XX germ cells (gKO). (**C**) The number of caspase3+ cells is significantly higher in the N-cadherin knockout in the XY and XX gonadal somatic cells (sKO) than in the control after E14.5. (**D**) The number of caspase3+ cells is significantly higher in the N-cadherin knockout in the XY and XX germ cells (gKO) than in the control after E14.5. * $P<0.05$ (by χ^2); three samples issued from the same developmental stage and corresponding to the same genotype were analyzed in each time point, thus in total 72 speciemens were used for this analysis.

Suppl. Fig. S7.

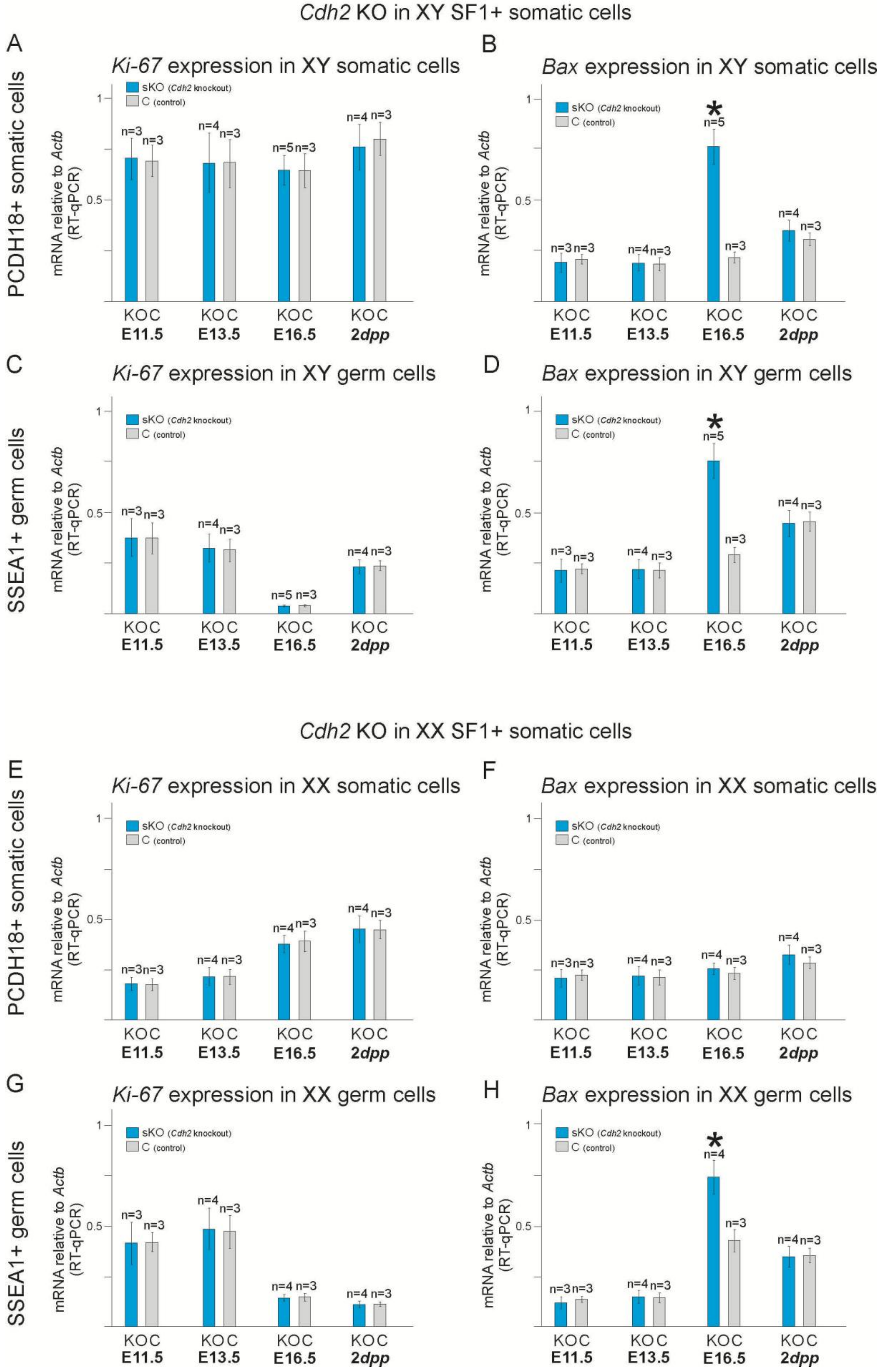

Cdh2 KO in XY SF1+ somatic cells
A
Ki-67 expression in XY somatic cells
PCDH18+ somatic cells
mRNA relative to Actb (RT-qPCR)
sKO (Cdh2 knockout)
C (control)
n=3 n=3 n=4 n=3 n=5 n=3 n=4 n=3
KO C E11.5 KO C E13.5 KO C E16.5 KO C 2dpp
B
Bax expression in XY somatic cells
mRNA relative to Actb (RT-qPCR)
sKO (Cdh2 knockout)
C (control)
*
n=3 n=3 n=4 n=3 n=5 n=3 n=4 n=3
KO C E11.5 KO C E13.5 KO C E16.5 KO C 2dpp
C
Ki-67 expression in XY germ cells
SSEA1+ germ cells
mRNA relative to Actb (RT-qPCR)
sKO (Cdh2 knockout)
C (control)
n=3 n=3 n=4 n=3 n=5 n=3 n=4 n=3
KO C E11.5 KO C E13.5 KO C E16.5 KO C 2dpp
D
Bax expression in XY germ cells
mRNA relative to Actb (RT-qPCR)
sKO (Cdh2 knockout)
C (control)
*
n=3 n=3 n=4 n=3 n=5 n=3 n=4 n=3
KO C E11.5 KO C E13.5 KO C E16.5 KO C 2dpp
Cdh2 KO in XX SF1+ somatic cells
E
Ki-67 expression in XX somatic cells
PCDH18+ somatic cells
mRNA relative to Actb (RT-qPCR)
sKO (Cdh2 knockout)
C (control)
n=3 n=3 n=4 n=3 n=4 n=3 n=4 n=3
KO C E11.5 KO C E13.5 KO C E16.5 KO C 2dpp
F
Bax expression in XX somatic cells
mRNA relative to Actb (RT-qPCR)
sKO (Cdh2 knockout)
C (control)
n=3 n=3 n=4 n=3 n=4 n=3 n=4 n=3
KO C E11.5 KO C E13.5 KO C E16.5 KO C 2dpp
G
Ki-67 expression in XX germ cells
SSEA1+ germ cells
mRNA relative to Actb (RT-qPCR)
sKO (Cdh2 knockout)
C (control)
n=3 n=3 n=4 n=3 n=4 n=3 n=4 n=3
KO C E11.5 KO C E13.5 KO C E16.5 KO C 2dpp
H
Bax expression in XX germ cells
mRNA relative to Actb (RT-qPCR)
sKO (Cdh2 knockout)
C (control)
*
n=3 n=3 n=4 n=3 n=4 n=3 n=4 n=3
KO C E11.5 KO C E13.5 KO C E16.5 KO C 2dpp

Supplementary Figure S7. Expression of *Ki-67* and *Bax* in the somatic and germ cells isolated from *Sf1-Cre+ Cdh2* knockout gonads. (**A**) *Ki-67* expression in the XY PCDH18^{+} cells. (**B**) *Bax* expression in the XY PCDH18^{+} cells – higher expression in the knockout testes. (**C**) *Ki-67* expression in the XY SSEA1^{+} cells. (**D**) *Bax* expression in the XY SSEA1^{+} cells – higher expression in the XY germ cells. (**E**) *Ki-67* expression in the XX PCDH18^{+} cells. (**F**) *Bax* expression in the XX PCDH18^{+} cells. (**G**) *Ki-67* expression in the XX SSEA1^{+} cells. (**H**) *Bax* expression in the XX SSEA1^{+} cells – higher expression in the XX germ cells. * $P<0.05$ (by χ^2), n – the number of individuals tested, deviation bars indicate standard deviation.

Suppl. Fig. S8.

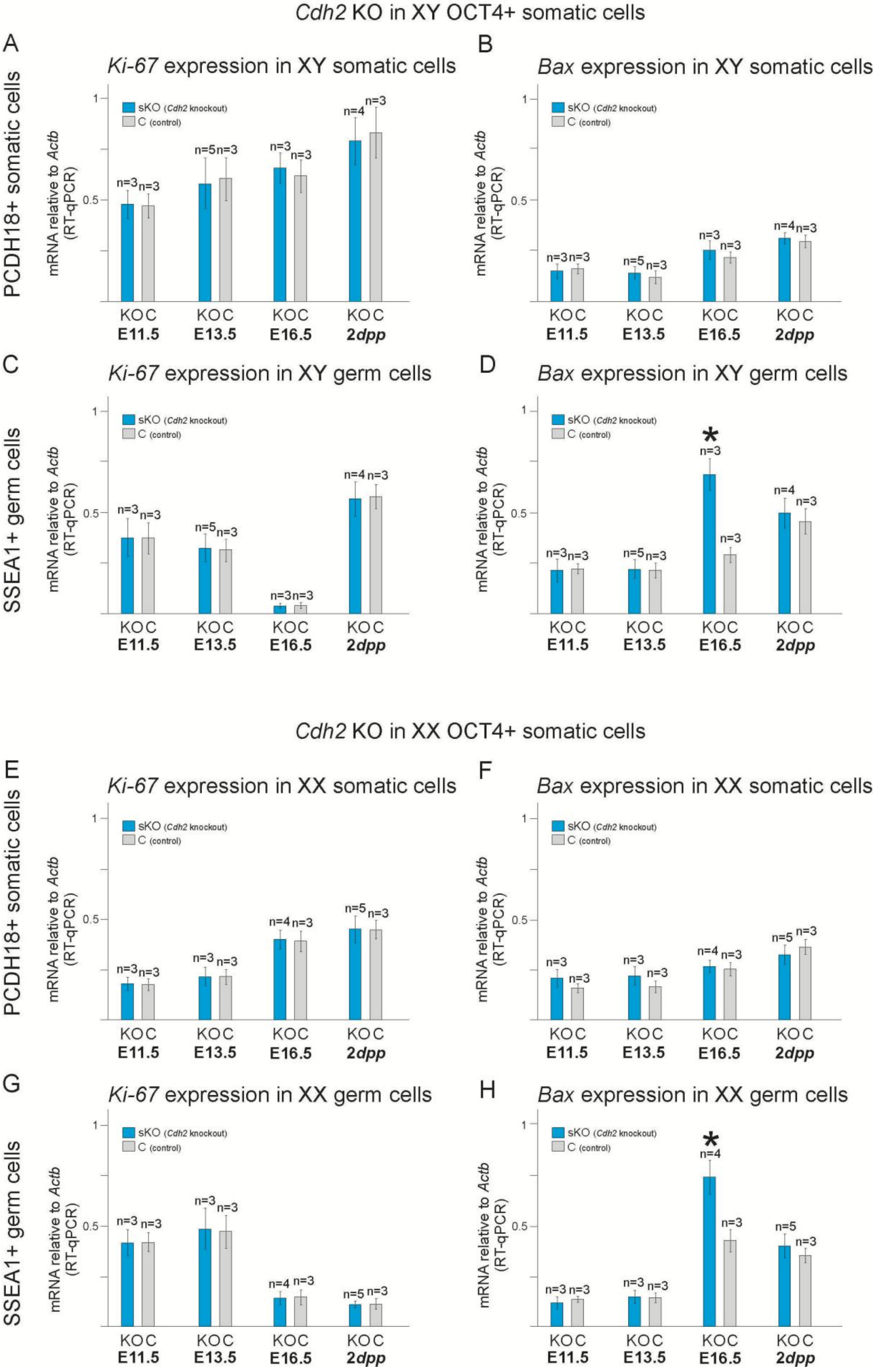
Cdh2 KO in XY OCT4+ somatic cells
A
Ki-67 expression in XY somatic cells
PCDH18+ somatic cells
mRNA relative to Actb (RT-qPCR)
sKO (Cdh2 knockout)
C (control)
n=3 n=3 n=5 n=3 n=3 n=3 n=4 n=3
KO C E11.5 KO C E13.5 KO C E16.5 KO C 2dpp
B
Bax expression in XY somatic cells
n=3 n=3 n=5 n=3 n=3 n=3 n=4 n=3
C
Ki-67 expression in XY germ cells
SSEA1+ germ cells
n=3 n=3 n=5 n=3 n=3 n=3 n=4 n=3
D
Bax expression in XY germ cells
n=3 n=3 n=5 n=3 * n=3 n=3 n=4 n=3
Cdh2 KO in XX OCT4+ somatic cells
E
Ki-67 expression in XX somatic cells
PCDH18+ somatic cells
n=3 n=3 n=3 n=3 n=4 n=3 n=5 n=3
F
Bax expression in XX somatic cells
n=3 n=3 n=3 n=3 n=4 n=3 n=5 n=3
G
Ki-67 expression in XX germ cells
SSEA1+ germ cells
n=3 n=3 n=3 n=3 n=4 n=3 n=5 n=3
H
Bax expression in XX germ cells
n=3 n=3 n=3 n=3 * n=4 n=3 n=5 n=3
1
0.5

Supplementary Figure S8. Expression of *Ki-67* and *Bax* in the somatic and germ cells isolated from *Oct4-Cre+ Cdh2* knockout gonads. (**A**) *Ki-67* expression in the XY $PCDH18^+$ cells. (**B**) *Bax* expression in the XY $PCDH18^+$ cells. (**C**) *Ki-67* expression in the XY $SSEA1^+$ cells. (**D**) *Bax* expression in the XY $SSEA1^+$ cells – higher expression in the XY germ cells. (**E**) *Ki-67* expression in the XX $PCDH18^+$ cells. (**F**) *Bax* expression in the XX $PCDH18^+$ cells. (**G**) *Ki-67* expression in the XX $SSEA1^+$ cells. (**H**) *Bax* expression in the XX SSEA1+ cells – higher expression in the XX germ cells. * $P<0.05$ (by χ^2), n – the number of individuals tested, deviation bars indicate standard deviation.

Suppl. Fig. 9.

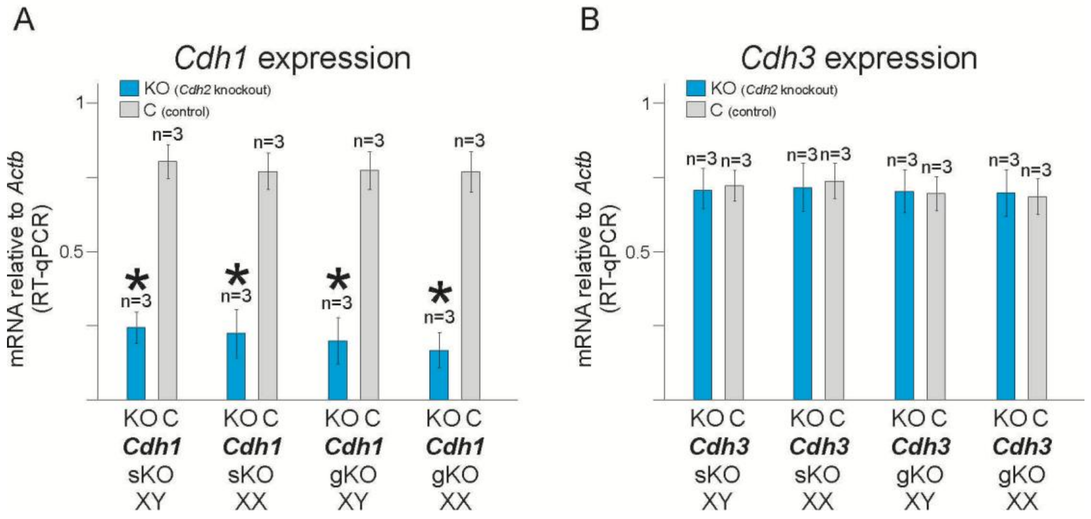

Supplementary Figure S9. Expression of *Cdh1* (E-cadherin) and *Cdh3* (P-cadherin) in the control and knockout gonads at E18.5. (**A**) The expression of *Cdh1* gene is significantly lower in the knockout XY and XX gonads than in the control. (**B**) There is no significant difference in the expression of *Cdh3* between the knockout and control gonads. * $P<0.05$ (by χ^2), n – the number of individuals tested, deviation bars indicate standard deviation.

Suppl. Table S1. Primers used for genotyping and real-time qPCR.

Gene	Primers	Gene function
Primers used for genotyping		
SX (sex genotyping)	F: GATGATTTGAGTGGAAATGTGAGGTA<br>R: CTTATGTTTATAGGCATGCACCATGTA	-
Oct4-cre	Common: CCAAGGCAAGGGAGGTAGACAAG<br>Wild type: GCTTTCTCCAACCGCAGGCTCTC<br>Mutant: GCCCTCACATTGCCAAAAGACGG	-
Sf1-cre	Transgene F: CTGAGCTGCAGCGCAGGGACAT<br>Transgene R: TGCGAACCTCATCACTCGTTGCAT<br>Control F: CAAATGTTGCTTGTCTGGTG<br>Control R: GTCAGTCGAGTGCACAGTTT	-
Cdh2-loxP	F: CCAAAGCTGAGTGTGACTTG<br>R: TACAAGTTTGGGTGACAAGC	-
Primers used for RT-qPCR		
Actb	F: CATGTACGTTGCTATCCAGGC<br>R: CTCCTTAATGTCACGCACGAT	Beta-actin – housekeeping gene
Amh	F: TCAACCAAGCAGAGAAGGTG<br>R: AGTCATCCGCGTGAAACAG	Anti-Müllerian hormone – Sertoli cell marker
Bax	F: GCGTGGTTGCCCTCTTCTACTTTG<br>R: AGTCCAGTGTCCAGCCCATGATG	Bcl-2-associated X protein – apoptosis marker
Cdh1	F: GGTTTTCTACAGCATCACCG<br>R: GCTTCCCCATTTGATGACAC	E-cadherin – adhesion protein
Cdh2	F: AACAGTCTCCAAGTGGCCAGG<br>R: TGGTAACAAATAGCGGGCCT	N-cadherin – adhesion protein
Cre	F: CCCTGTTTCACTATCCAGGT<br>R: GGGTAACTAAACTGGTCGAG	Cre recombinase
Cyp11a1	F: GTGAATGACCTGGTGCTTGGT<br>R: TCGACCCATGGCAAAGCTA	Cholesterol side-chain cleavage enzyme – steroidogenic cell marker

Suppl. Table S1. Primers used for genotyping and real-time qPCR, continuation.

Gene	Primers	Gene function
Primers used for RT-qPCR		
Dazl	F: TGGACCGAAGCATACAGACAGTGGT<br>R: CACCGTCATGGTCTTTGTAGTC	Deleted in azoospermia-like – germ cell marker
Dhh	F: TGATGACCGAGCGTTGTAAG<br>R: GCCAGCAACCCATACTTGTT	Desert hedgehog – fetal Leydig cell marker and marker of testis differentiation
Foxl2	F: GCTACCCCGAGCCCGAAGAC<br>R: GTGTTGTCCCGCCTCCCTTG	Forkhead box L2 – gene involved in female sex determination
Ki-67	F: AATCCAACTCAAGTAAACGGGG<br>R: TTGGCTTGCTTCCATCCTCA	Proliferation marker protein Ki67
Mvh	F: GAGATTGCCTTCAGTACCTATGTG<br>R: GTGCTTGCCCTGGTAATTCT	Mouse vasa homolog – germ cell marker
Rn18s	F: GATCCATTGGAGGGCAAGTCT<br>R: CCAAGATCCAACTACGAGCTTTTT	18S ribosomal RNA – house keeping gene
Rspo1	F: TGTGAAATGAGCGAGTGGTCC<br>R: TCTCCCAGATGCTCCAGTTCT	R-spondin 1 – gene involved in female sex determination
Sox9	F: GTGCAAGCTGGCAAAGTTGA<br>R: TGCTCAGTTCACCGATGTCC	SRY (sex determining region Y)-box 9 – gene involved in male sex determination
Wnt4	F: TGTACCTGGCCAAGCTGTCAT<br>R: TCCGGTCACAGCCACACTT	Wingless-Type MMTV Integration Site Family, Member 4 – gene involved in female sex determination

Suppl. Table S2. Number of specimens used for real-time qPCR analysis.

	Genotype	E10.5		E11.5		E12.5		E13.5		E14.5		E16.5		E18.5		1*dpp*		2*dpp*		Sum
		XX	XY	XX	XY	XX	XY	XX	XY	XX	XY	XX	XY	XX	XY	XX	XY	XX	XY	
Knockout of N-cadherin in somatic cells	**Sf11-Cre+ Cdh2+/+ control**	3	3	3	3	-	-	3	3	-	-	6	6	9	9	-	-	3	3	54
	**Sf11-Cre+ Cdh2fl/fl knockout**	3	3	3	3	-	-	4	4	-	-	9	11	9	9	-	-	4	4	66
Knockout of N-cadherin in germ cells	**Oct4-Cre+ Cdh2+/+ control**	3	3	3	3	-	-	3	3	-	-	6	9	9	9	-	-	3	3	57
	**Oct4-Cre+ Cdh2fl/fl knockout**	3	3	3	3	-	-	3	5	-	-	10	10	9	9	-	-	5	4	67

Suppl. Table S3. Number of specimens used for histological analysis.

	Genotype	E10.5		E11.5		E12.5		E13.5		E14.5		E16.5		E18.5		1*dpp*		2*dpp*		Sum
		XX	XY	XX	XY	XX	XY	XX	XY	XX	XY	XX	XY	XX	XY	XX	XY	XX	XY	
Knockout of N-cadherin in somatic cells	**Sf11-Cre+ Cdh2+/+ control**	5	4	7	6	4	4	7	6	4	4	4	5	10	10	10	9	10	11	120
	**Sf11-Cre+ Cdh2fl/fl knockout**	5	6	6	5	5	5	8	9	4	5	4	5	11	12	11	11	11	10	133
Knockout of N-cadherin in germ cells	**Oct4-Cre+ Cdh2+/+ control**	5	4	6	6	4	4	6	7	4	5	4	6	8	8	8	9	10	11	115
	**Oct4-Cre+ Cdh2fl/fl knockout**	6	6	5	6	6	5	8	9	4	5	6	7	8	10	10	9	11	11	132
